# A WHY1–NADK3–PEX5 module confers drought tolerance by maintaining peroxisomal ROS homeostasis in *Brassica napus*

**DOI:** 10.1111/tpj.71124

**Published:** 2026-09-30

**Authors:** Jiaqi Yan, Zhaoyang Qu, Chao Wang, Ze Tian, Lieqiong Kuang, Yu Mu, Hanzhong Wang, Xiaoling Dun

**Affiliations:** ^1^ College of Agronomy Northwest A&F University Yangling China; ^2^ Key Laboratory of Biology and Genetic Improvement of Oil Crops, Oil Crops Research Institute of the Chinese Academy of Agricultural Sciences Ministry of Agriculture and Rural Affairs Wuhan China; ^3^ Hubei Hongshan Laboratory Wuhan China

**Keywords:** *Brassica napus*, drought tolerance, NADPH, ROS homeostasis, *BnaA07.NADK3*, *BnaA03.PEX5*

## Abstract

Drought severely limits rapeseed productivity, but the role of NAD kinase in this process remains unclear. Here, we identify BnaA07.NADK3 as a drought‐ and ABA‐inducible peroxisomal NADK in *Brassica napus*. Overexpression of BnaA07.NADK3 enhanced drought tolerance, whereas CRISPR/Cas9‐edited lines showed reduced drought tolerance. Mechanistically, overexpression of *BnaA07.NADK3* elevates NADK activity and NADPH accumulation, thereby promoting the scavenging of drought‐induced reactive oxygen species (ROS). Transcriptome profiling of *BnaA07.NADK3*‐overexpressing plants revealed significant enrichment of genes involved in ROS detoxification and root development. Both *in vivo* and *in vitro* assays demonstrate that BnaA07.NADK3 physically interacts with the peroxisomal import receptor BnaA03.PEX5 (Peroxin 5), suggesting that BnaA07.NADK3 is associated with the peroxisomal protein import machinery required for maintaining ROS homeostasis under stress. In addition, BnaC08.WHY1 directly binds a conserved TTTTTGAC element in the *BnaA07.NADK3* promoter and activates its transcription. In summary, our findings uncover a previously unrecognized WHY1–NADK3–PEX5 regulatory module that integrates plastid retrograde signaling, peroxisomal redox metabolism, and drought adaptation in *Brassica napus*, providing promising targets for molecular breeding of drought‐tolerant rapeseed cultivars.

## Significance Statement

Drought tolerance depends on maintaining redox balance, but how stress signaling is connected with NADPH metabolism in rapeseed remains unclear. We show that BnaC08.WHY1 activates *BnaA07.NADK3* transcription, while BnaA07.NADK3 promotes NADPH accumulation, limits ROS accumulation, and interacts with BnaA03.PEX5, identifying a regulatory connection that contributes to ROS homeostasis and drought tolerance under stress.

## INTRODUCTION

Drought is a major constraint on crop productivity and is becoming increasingly severe under ongoing climate change. A central consequence of drought stress is the disruption of cellular redox balance, which leads to excessive accumulation of reactive oxygen species (ROS) and imposes strong metabolic constraints on plant growth and survival (Zhang, Zhu, et al., 2022). Improving the capacity of crops to maintain redox homeostasis under drought is therefore critical for sustainable agricultural production.

Nicotinamide adenine dinucleotide phosphate [NADP(H)] is a core redox cofactor that supports antioxidant metabolism, redox signaling, and multiple biosynthetic processes in plants (Goodman et al., 2018; Xiao et al., 2018). NAD kinases (NADKs), the only known enzymes that phosphorylate NAD(H) to generate NADP(H), are therefore central to cellular redox regulation (Ji et al., 2022). Plant NADKs regulate NADP(H) homeostasis and have been implicated in redox balance, ROS homeostasis, ABA‐associated stress responses, chloroplast function, and peroxisome‐associated metabolism (Chai et al., 2006; Chaomurilege et al., 2023; Ji et al., 2022; Li et al., 2018; Lu et al., 2025; Suzuki et al., 2023). However, the isoform‐specific functions and regulatory mechanisms of NADKs, particularly in crops, remain incompletely understood.

In *Arabidopsis thaliana*, NADK1, NADK2, and NADK3 exhibit distinct subcellular localizations, with NADK1 in the cytosol, NADK2 in the plastid stroma, and NADK3 in the peroxisomal matrix (Waller et al., 2010). NADK2 has been implicated in chlorophyll synthesis, chloroplast protection, photosynthetic energy transduction, and carbon/nitrogen metabolism (Chai et al., 2005; Takahashi et al., 2006, 2009). NADK3, another member of the plant NADK family, is biochemically distinct in that it preferentially phosphorylates NADH to produce NADPH (Turner et al., 2005). The physiological relevance of NADK3 is further supported by the hypersensitivity of *nadk3* mutants to abscisic acid (ABA) and mannitol, suggesting that NADK3 participates in abiotic stress adaptation (Chai et al., 2006). However, how NADK3 is transcriptionally activated under stress and targeted to peroxisomes remains unclear.

Efficient stress adaptation requires coordination between stress‐responsive transcriptional regulation and organelle function (Zhao et al., 2020). WHIRLY1 (WHY1), a plant‐specific DNA/RNA‐binding protein localized to both chloroplasts and the nucleus, has been implicated in redox‐associated stress signaling and stress‐responsive gene expression (Grabowski et al., 2008; Lin et al., 2020; Maréchal & Brisson, 2010; Taylor et al., 2023). Under drought conditions, WHY1 shows enhanced nuclear accumulation, consistent with a role in stress‐responsive transcriptional regulation. Peroxin 5 (PEX5) is a cytosolic receptor required for the import of peroxisomal matrix proteins carrying a peroxisomal targeting signal type 1 (PTS1) in plants (Kao et al., 2018; Khan & Zolman, 2010; Zolman et al., 2000). In *Arabidopsis*, *pex5* mutations impair peroxisomal matrix protein import and disrupt peroxisome‐dependent metabolic processes, including indole‐3‐butyric acid metabolism and fatty acid β‐oxidation (Khan & Zolman, 2010). Despite their individual importance, it remains unknown whether WHY1‐dependent transcriptional regulation and PEX5‐associated protein targeting converge on NADK3 to coordinate peroxisomal ROS homeostasis during drought adaptation.

Rapeseed (*Brassica napus* L.), one of the world's major oil crops, is highly vulnerable to drought and other abiotic stresses, making improved stress tolerance a major breeding objective (Tan et al., 2024). Recent advances in CRISPR/Cas9‐based mutant resources and integrated genetic and transcriptomic analyses have facilitated functional gene discovery and trait improvement in *B. napus* (Wu et al., 2026; Zeng et al., 2026). Although *NADK* family members have been implicated in abiotic stress responses in model species (Chai et al., 2006; Ji et al., 2022; Suzuki et al., 2023), their functions and regulatory networks in *B. napus* remain poorly defined. In this study, we identify *BnaA07.NADK3* as a drought‐ and ABA‐responsive peroxisomal NADK in rapeseed. *BnaA07.NADK3* promotes drought tolerance by enhancing NADPH accumulation and ROS detoxification capacity. We further show that *BnaC08.WHY1* activates *BnaA07.NADK3* transcription and that BnaA07.NADK3 functionally associates with the peroxisomal receptor BnaA03.PEX5 to support peroxisomal ROS homeostasis under drought stress. These findings define a WHY1–NADK3–PEX5 module underlying drought adaptation in *B. napus*.

## RESULTS

### *BnaA07.NADK3* is induced by drought and ABA and localizes to peroxisomes

Although members of the NAD kinase (NADK) family are known to be involved in plant development and stress responses (Chai et al., 2006; Li et al., 2018; Suzuki et al., 2023), their specific functions in *Brassica napus* remain largely unexplored. To investigate NADK members associated with stress adaptation, we first identified 12 *BnaNADK* genes within the *B. napus* genome (Figure S1A). Subsequent RNA sequencing (RNA‐seq) analysis of seedlings subjected to various abiotic stresses (ABA, PEG, heat, NaCl, and cold) revealed that *BnaA07.NADK3* exhibited the most prominent transcriptional induction (Figure S1B). Notably, its expression was markedly upregulated in root tissues following ABA and PEG treatments (Figure 1A). These transcriptomic profiles were further validated via quantitative real‐time PCR (RT‐qPCR), which confirmed that *BnaA07.NADK3* expression in roots was significantly induced by both ABA and PEG‐simulated drought treatments, peaking at 24‐h post‐treatment (Figure 1B). Collectively, these findings suggest that *BnaA07.NADK3* is highly responsive to abiotic stress and potentially plays a crucial role in drought adaptation in *B. napus*.

**Figure 1:**
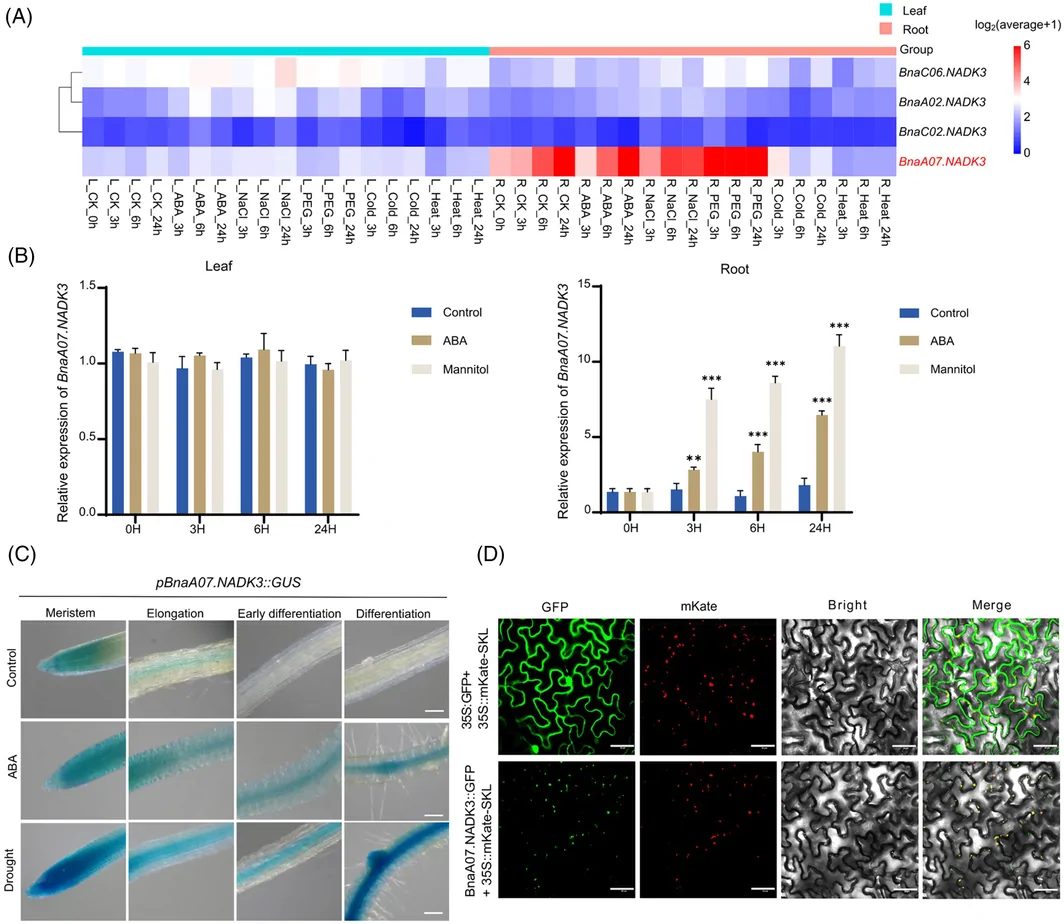
Expression pattern and subcellular localization of *BnaA07.NADK3*. (A) Expression profiles of the four *BnaNADK3* genes under different abiotic stresses. Data are a subset of the RNA‐seq dataset shown in Figure S1B. (B) RT‐qPCR analysis of *BnaA07.NADK3* expression in leaves and roots under ABA and mannitol treatments (*n* = 3 biological replicates). Asterisks indicate statistically significant differences (**P < 0.01, ***P < 0.001; Student's *t*‐test). (C) GUS staining of Pro*BnaA07.NADK3::GUS* transgenic hairy roots under control, ABA, and drought conditions. Scale bars = 200 μm. (D) Subcellular localization of BnaA07.NADK3. GFP–BnaA07.NADK3 and the GFP control were transiently co‐expressed with the peroxisomal marker mKate–SKL in *Nicotiana benthamiana* leaf epidermal cells. GFP and mKate signals are shown in green and red, respectively. Signal overlap is shown in yellow in the merged images. Scale bars = 20 μm.

To further investigate the function of *BnaA07.NADK3*, we constructed the transcriptional fusion construct Pro*BnaA07.NADK3::GUS* and introduced it into *B. napus* via hairy root transformation. Histochemical GUS staining revealed enhanced signals under ABA and drought stress conditions, further confirming that *BnaA07.NADK3* is induced by both drought and ABA (Figure 1C). AtNADK3 has previously been reported to localize to peroxisomes in *Arabidopsis* (Waller et al., 2010). To determine whether BnaA07.NADK3 showed a similar localization, GFP–BnaA07.NADK3 was transiently co‐expressed with mKate fused to the C‐terminal peroxisomal targeting signal 1 (PTS1) tripeptide Ser‐Lys‐Leu (mKate–SKL), which served as a peroxisomal marker (Mano et al., 2002; Nelson et al., 2007), in *Nicotiana benthamiana* leaf epidermal cells. GFP–BnaA07.NADK3 showed a punctate distribution and largely colocalized with mKate–SKL, whereas GFP alone did not colocalize with the peroxisomal marker. These results support the peroxisomal localization of BnaA07.NADK3 (Figure 1D). Consequently, these findings indicate that BnaA07.NADK3 is a peroxisome‐localized protein whose expression is strongly induced by drought and ABA, suggesting its potential involvement in peroxisome‐associated metabolic pathways that mediate abiotic stress adaptation in *B. napus*.

### *BnaA07.NADK3* enhances drought tolerance by promoting ROS scavenging in *B. napus*

To investigate the role of *BnaA07.NADK3* in ABA responses and drought tolerance, we generated *B. napus* lines overexpressing *BnaA07.NADK3* (OE) and two independent CRISPR/Cas9‐edited lines, designated KO‐1 and KO‐2, and compared their phenotypes with those of wild‐type (WT) plants (Figure S2A,C). Under normal growth conditions on 1/2 MS medium, no significant phenotypic differences were observed among the transgenic lines and WT. However, after exposure to 100 μm ABA or 200 mm mannitol for 7 days, OE lines exhibited enhanced stress tolerance, as evidenced by reduced inhibition of primary root elongation and higher fresh weight compared to WT. In contrast, the CRISPR/Cas9‐edited lines were more sensitive under both treatments, exhibiting shorter primary roots and lower biomass than WT plants (Figure S3A–C).

Biochemical analyses revealed that OE plants exhibited significantly higher activities of major ROS‐scavenging enzymes, namely superoxide dismutase (SOD), catalase (CAT), and peroxidase (POD), under stress conditions, accompanied by reduced malondialdehyde (MDA) content, indicating mitigated oxidative damage. Conversely, the CRISPR/Cas9‐edited lines showed significantly decreased antioxidant enzyme activities and increased MDA accumulation compared with WT plants (Figure S3D–G).

To assess drought tolerance under more physiologically relevant conditions, soil‐grown plants were subjected to water withholding for 2 weeks, followed by rewatering for 7 days (Figure 2). Consistent with the agar culture test results, OE lines maintained higher CAT, SOD, and POD activities and lower MDA contents during the drought treatment, whereas CRISPR/Cas9‐edited lines showed the opposite pattern (Figure 2C–F). Compared with WT, OE plants showed significantly higher survival rates, longer primary roots, greater root fresh weight, and higher shoot biomass, whereas the gene‐edited lines performed worse, displaying reduced survival, shorter primary roots, and lower root fresh weight and shoot biomass (Figure 2G–J). Collectively, these results indicate that BnaA07.NADK3 promotes drought tolerance in *B. napus* by enhancing ROS‐scavenging capacity and mitigating oxidative damage. In addition, RT‐qPCR analysis of representative ABA‐responsive genes showed higher transcript levels in the OE lines and lower levels in the CRISPR/Cas9‐edited lines than in WT plants following ABA treatment (Figure S4). These genotype‐dependent expression patterns are consistent with a role for *BnaA07.NADK3* in modulating ABA responses.

**Figure 2:**
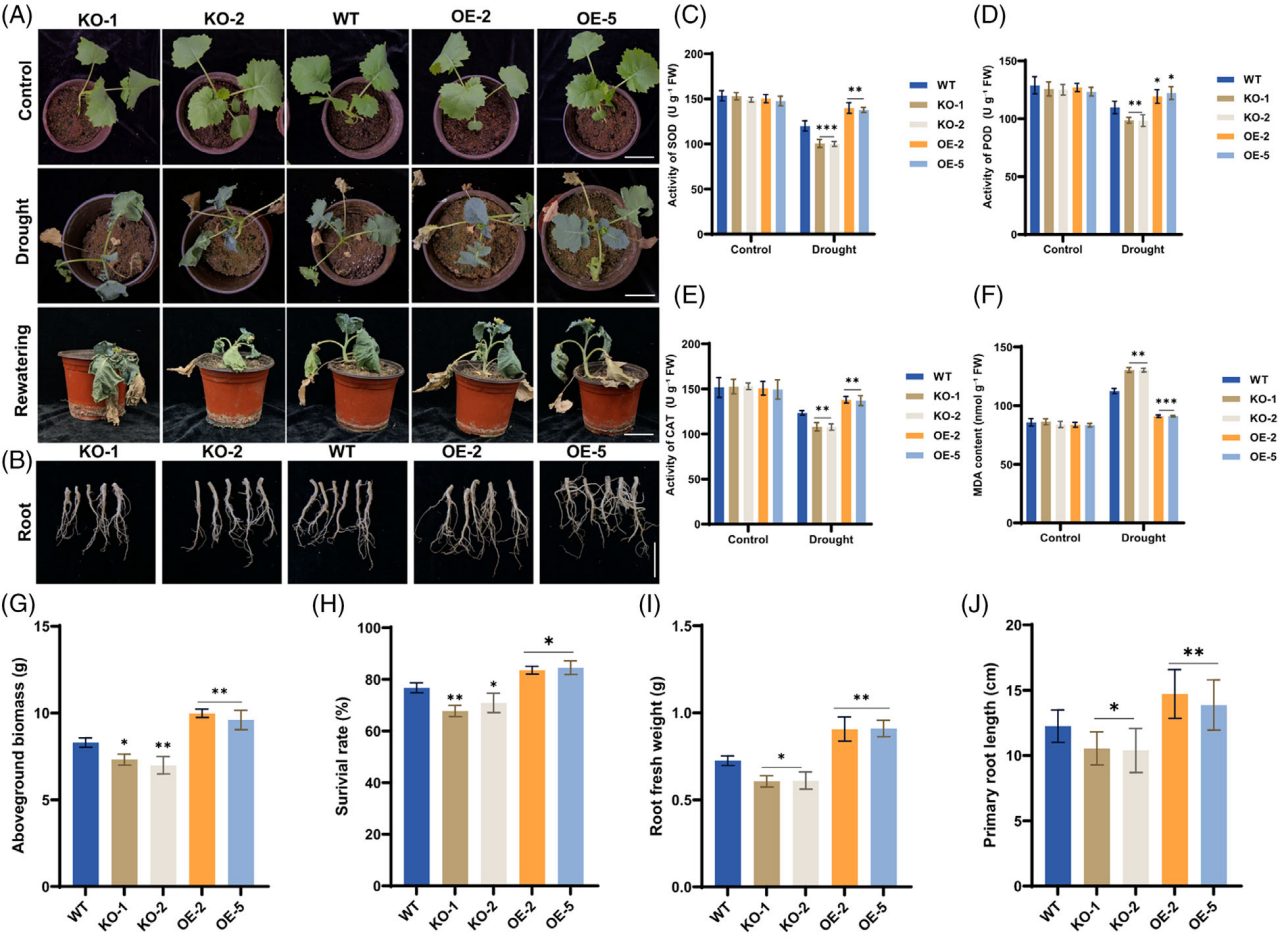
*BnaA07.NADK3* enhances drought tolerance and antioxidant capacity in *Brassica napus*. (A, B) Representative phenotypes and root architecture of WT, *BnaA07.NADK3* CRISPR/Cas9‐edited lines (KO‐1 and KO‐2), and overexpression lines (OE‐2 and OE‐5) under control conditions, drought treatment, and rewatering. Plants were subjected to drought stress for 2 weeks and then rewatered for 1 week. Scale bars = 7 cm. (C–F) Activities of superoxide dismutase (SOD), peroxidase (POD), and catalase (CAT), and malondialdehyde (MDA) content in WT plants, CRISPR/Cas9‐edited lines, and OE lines under control and drought conditions. (G–J) Aboveground biomass, survival rate, root fresh weight, and primary root length of WT plants, CRISPR/Cas9‐edited lines, and OE lines in the drought assay. Data are presented as mean ± SD (*n* = 3 biological replicates; 15 plants per genotype per replicate). Data were analyzed by one‐way anova followed by Dunnett's multiple comparisons test. Asterisks indicate significant differences compared with WT under the same treatment (*P < 0.05, **P < 0.01, ***P < 0.001).

### *BnaA07.NADK3* mediates temporal ROS‐scavenging responses under stress

To elucidate the transcriptional landscape governed by *BnaA07.NADK3* under abiotic stress, we performed RNA‐seq using root tissues from plate‐grown OE and WT seedlings. Seedlings were grown on 1/2 MS agar plates and transferred to 1/2 MS agar plates containing ABA or mannitol, with untreated 1/2 MS agar plates used as the control. Root tissues were collected at 0 h, 6 h, and 1 week after treatment. Differentially expressed genes (DEGs) were identified for each OE‐versus‐WT comparison under the respective treatments using thresholds of FDR ≤0.05 and |log_2_FC| ≥ 1. To dissect the *BnaA07.NADK3*‐dependent stress‐responsive transcriptional changes, we focused on DEGs that were simultaneously detected under both ABA and mannitol treatments, while excluding genes differentially expressed under control conditions (Figure 3A,B). At 6‐h post‐treatment, 1325 genes commonly upregulated and 1453 commonly downregulated genes were identified in OE roots relative to WT under both stress conditions. After 1 week, the number of shared DEGs markedly decreased to 366 upregulated and 73 downregulated genes, indicating a temporal attenuation and refinement of the transcriptional response.

**Figure 3:**
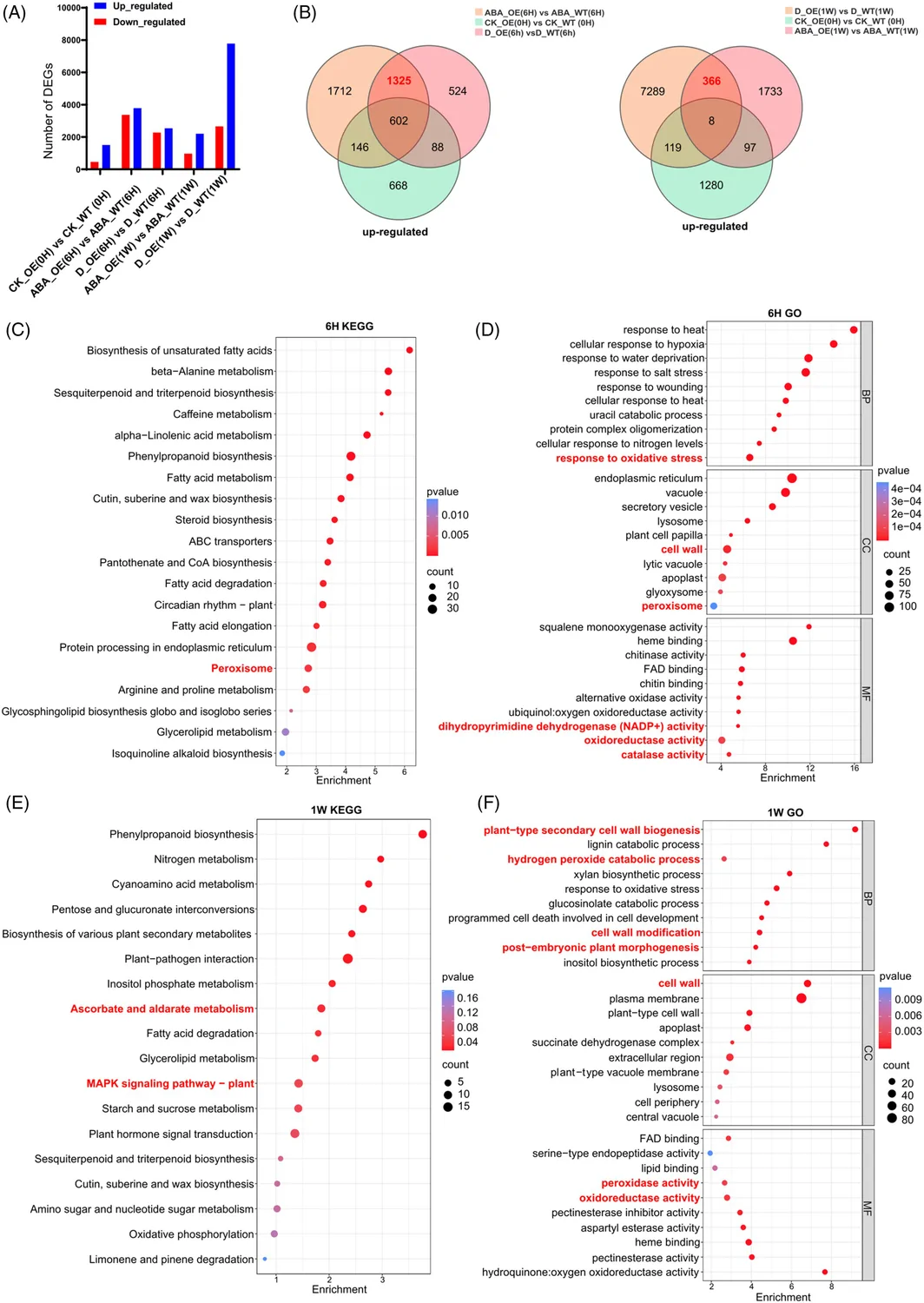
Comparative transcriptomic profiling of OE‐*BnaA07.NADK3* and WT plants under ABA and mannitol treatments. (A) Statistics of DEGs at 0 h, 6 h, and 1‐week post‐treatment. (B) Venn diagrams showing the overlap of upregulated differentially expressed genes (DEGs) between OE and WT plants under control (0 h), ABA, and mannitol treatments at 6 h and 1 week. (C, D) KEGG pathway and GO enrichment analyses of the overlapped upregulated DEGs under ABA and mannitol treatments at 6 h. (E, F) KEGG pathway and GO enrichment analyses of the overlapped upregulated DEGs under ABA and mannitol treatments at 1 week.

GO and KEGG enrichment analyses of these stage‐specific shared DEGs revealed distinct functional signatures. At 6 h, co‐upregulated genes were significantly enriched in pathways related to immediate redox homeostasis and structural defense, including peroxisome organization, response to oxidative stress, catalase activity, and cell wall components (Figure 3C,D). These enrichments suggest *BnaA07.NADK3* overexpression primes an early transcriptional enhancement of organelle‐associated antioxidant systems and apoplastic defense processes in roots.

In contrast, at 1 week, the co‐upregulated gene set was predominantly enriched in pathways associated with sustained metabolic adaptation, such as ascorbate and aldarate metabolism, MAPK signaling pathway, and plant‐type secondary cell wall biogenesis (Figure 3E,F). This temporal shift from direct enzymatic ROS scavenging to broader metabolic and structural reinforcement indicates a staged transcriptional program that transitions from acute stress response to long‐term acclimation. Conversely, the shared downregulated DEGs across both time points were primarily enriched in plant hormone signal transduction pathways and chloroplast‐related components, whereas no significant enrichment was detected for categories associated with ROS scavenging or root development (Figure S5C–F), suggesting a metabolic shift toward stress adaptation at the expense of growth‐related processes.

To validate these findings, we examined the expression of antioxidant‐ and redox‐associated genes, including genes encoding monodehydroascorbate reductase 1 (*MDAR1*) and dehydroascorbate reductase 1 (*DHAR1*), which are involved in ascorbate regeneration (Foyer & Kunert, 2024; Terai et al., 2020); the class III peroxidases peroxidase 11 (*PER11*) and peroxidase 44 (*PER44*), which are associated with ROS metabolism and apoplastic redox regulation (Marzol et al., 2022; Yu et al., 2016); the cell wall‐related redox genes SKU5 SIMILAR 13 (*SKS13*) and LACCASE 5 (*LAC5*) (Blaschek et al., 2023; Chen et al., 2023; Zhang, Zhao, et al., 2022); and the stress‐responsive cytochrome P450 gene CYTOCHROME P450, FAMILY 81, SUBFAMILY D, POLYPEPTIDE 11 (*CYP81D11*) (Tiwari et al., 2025). Transcriptome analysis showed that these genes were significantly upregulated in *BnaA07.NADK3*‐overexpressing lines under stress conditions (Figure S6A–D). RT‐qPCR further confirmed their consistently higher expression in OE lines following ABA and mannitol treatments. Collectively, these results indicate that BnaA07.NADK3 contributes to coordinated and stage‐specific transcriptional responses that promote ROS scavenging and redox homeostasis under stress, providing a molecular link between NADPH metabolism, root antioxidant capacity, and stress adaptation.

### BnaA07.NADK3 promotes ROS scavenging by increasing NADPH accumulation

Given the established role of NADK3 in generating NADPH within peroxisomes to maintain redox homeostasis (Chai et al., 2006), and the function of ROS as signaling molecules under ABA‐mediated drought responses (Watkins et al., 2017), we investigated whether BnaA07.NADK3 contributes to ROS metabolism by modulating NADPH production.

We first examined ROS accumulation in overexpression lines and CRISPR/Cas9‐edited lines under stress conditions. Histochemical staining with nitroblue tetrazolium (NBT) and 3,3′‐diaminobenzidine (DAB) showed that OE lines accumulated markedly less superoxide anion ($O_{2}^{\cdot -}$) and hydrogen peroxide (H_2_O_2_) in both roots and leaves under ABA and mannitol treatments, while the CRISPR/Cas9‐edited lines exhibited increased ROS accumulation compared to WT plants (Figure S7A–H). These observations were further supported by H_2_DCFDA staining, which quantitatively assessed ROS levels. Under normal growth conditions, wild‐type and transgenic plants exhibited no significant differences. However, following ABA or mannitol‐induced osmotic stress, OE lines showed significantly lower fluorescence intensity compared to WT; the CRISPR/Cas9‐edited lines showed elevated ROS signals (Figure 4A–D). We further assessed ROS accumulation in leaves of soil‐grown plants exposed to soil drying. Under well‐watered conditions, NBT and DAB staining intensities did not differ significantly among genotypes. After 7 days of soil drying, staining was stronger in KO‐1 and KO‐2 and weaker in OE‐2 and OE‐5 than in WT (Figure S8).

**Figure 4:**
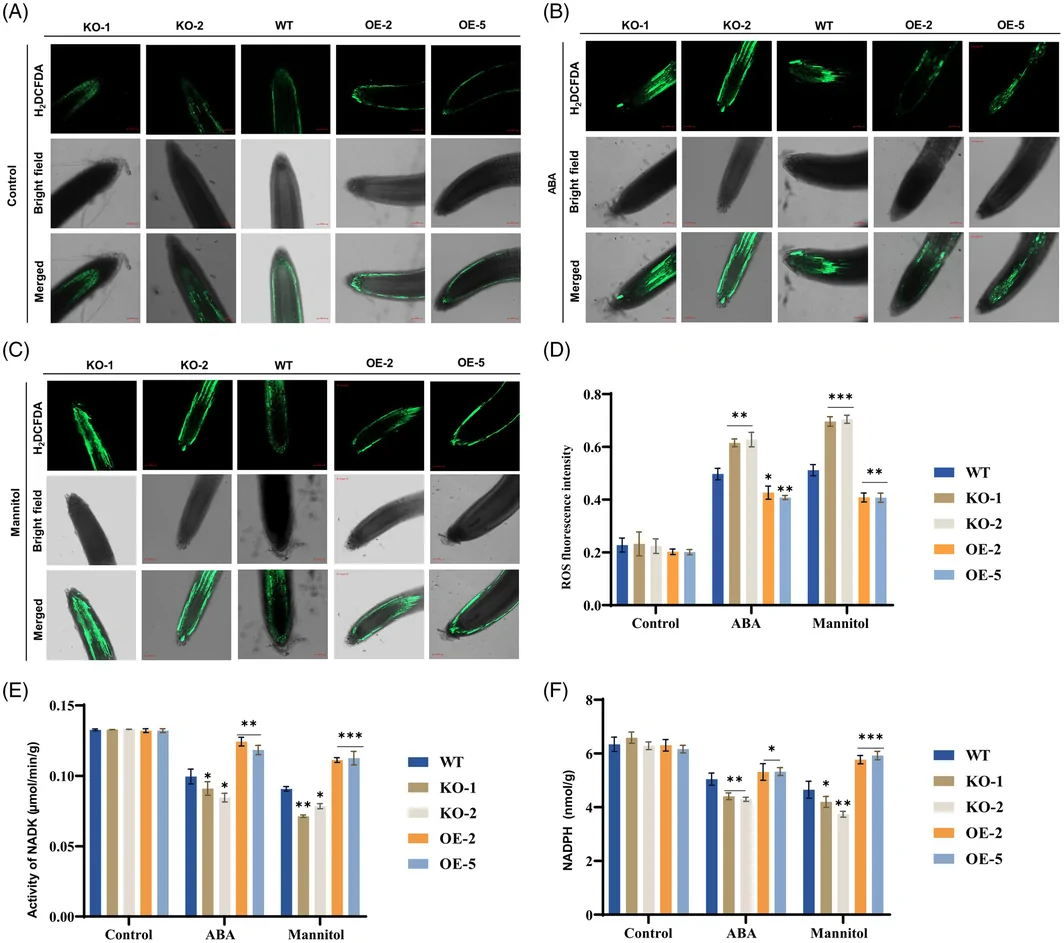
BnaA07.NADK3 enhances ROS scavenging by promoting NADPH accumulation under ABA and osmotic stress in *B. napus*. (A–C) Confocal laser scanning micrographs of H_2_DCFDA‐stained roots from WT, OE, and CRISPR/Cas9‐edited lines under control conditions and following ABA or mannitol treatment, showing the spatial distribution of ROS in roots (Scale bars = 200 μm). (D) Quantification of ROS‐associated fluorescence intensity. (E, F) NADK activity (E) and NADPH content (F) in WT, *BnaA07.NADK3* OE and CRISPR/Cas9‐edited lines. Data are presented as mean ± SD (*n* = 3 biological replicates; 5 plants per genotype per replicate). Data were analyzed by one‐way anova followed by Dunnett's multiple comparisons test. Asterisks indicate significant differences compared with WT under the same treatment (*P < 0.05, **P < 0.01, ***P < 0.001).

To determine whether the enhanced ROS‐scavenging capacity in OE lines resulted from increased NADPH production, we measured NADK activity and NADPH levels. Both NADPH levels and NADK activity were significantly elevated in OE lines, whereas they were notably reduced in the CRISPR/Cas9‐edited lines compared to WT plants (Figure 4E,F), indicating that BnaA07.NADK3 contributes to NADPH production and redox balance. These results confirm that BnaA07.NADK3 positively regulates ROS detoxification by enhancing NADK activity and NADPH accumulation, thereby strengthening the antioxidant defense system and contributing to improved drought tolerance in *B. napus*.

### BnaA07.NADK3 interacts with BnaA03.PEX5

To examine potential interaction partners of BnaA07.NADK3 involved in ROS regulation, we performed an electronic library‐based screen and identified BnaA03.PEX5 as a high‐confidence candidate (Table S1). Given that peroxisomal proteins are synthesized in the cytoplasm and must be transported into peroxisomes via specific receptors such as PEX5 and PEX7 (Hu et al., 2012), we hypothesized that BnaA03.PEX5 may facilitate the peroxisomal targeting of BnaA07.NADK3. We first validated their interaction using yeast two‐hybrid (Y2H) assays, which confirmed that BnaA07.NADK3 physically associates with BnaA03.PEX5 (Figure 5A). Additionally, in vitro MBP‐pulldown assays confirmed the direct interaction between MBP‐BnaA07.NADK3 and His‐BnaA03.PEX5, whereas the MBP negative control showed no binding (Figure 5B). The recombinant MBP‐BnaA07.NADK3 and His‐BnaA03.PEX5 proteins used in the pull‐down assays were purified and examined by Coomassie Brilliant Blue‐stained SDS‐PAGE (Figure S9). Bimolecular fluorescence complementation (BiFC) assays further demonstrated that this interaction occurs specifically within peroxisomes (Figure 5C). Luciferase complementation imaging (LCI) assays demonstrated that luminescence signals were detected only when both 1300nLUC‐BnaA07.NADK3 and 1300cLUC‐BnaA03.PEX5 were co‐expressed in tobacco leaves (Figure 5D). These results demonstrate that BnaA07.NADK3 interacts with BnaA03.PEX5 both *in vitro* and *in vivo*.

**Figure 5:**
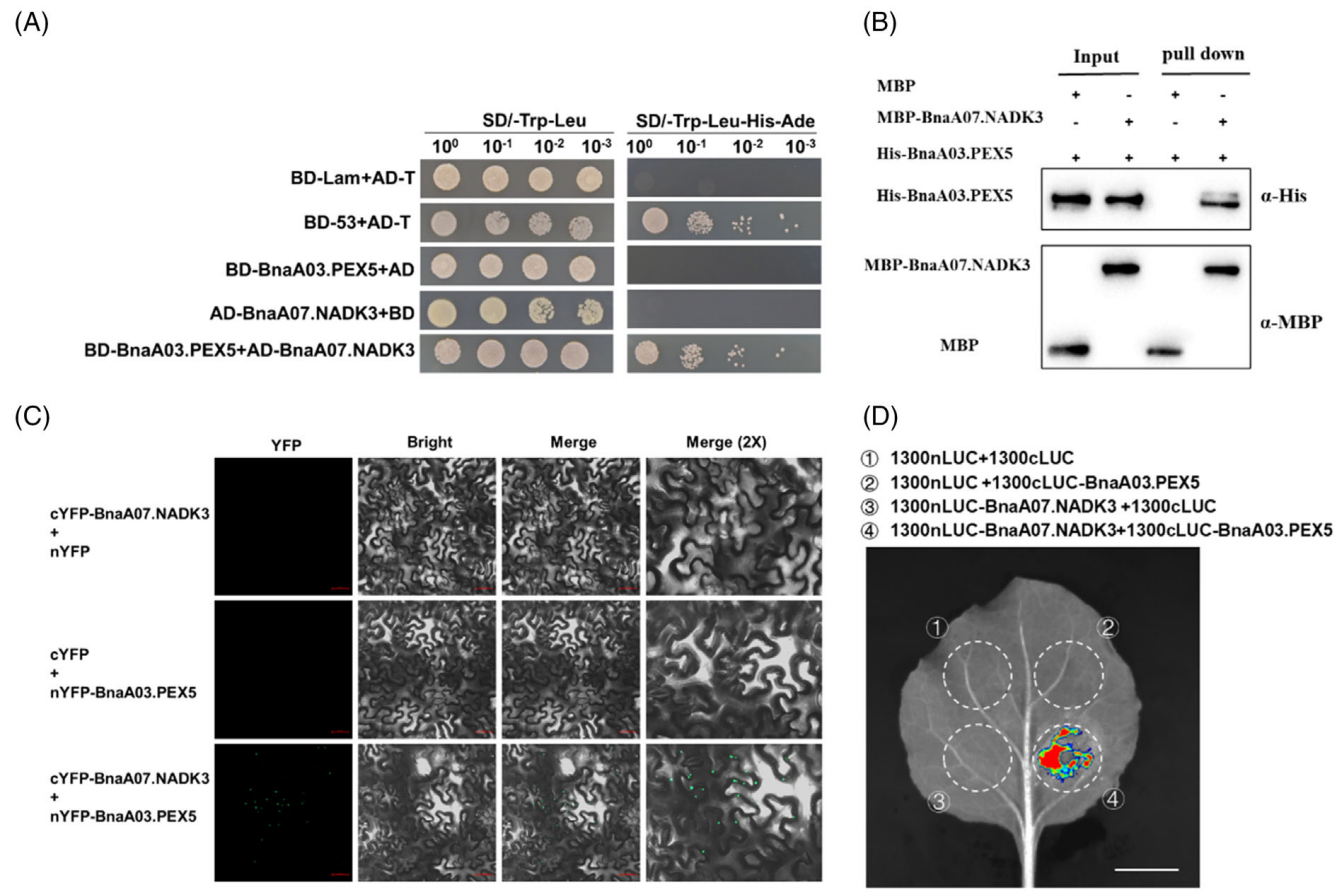
BnaA07.NADK3 interacts with BnaA03.PEX5 *in vitro* and *in vivo*. (A) Verification of the interaction of BnaA07.NADK3 with BnaA03.PEX5 by Y2H assays. Yeast transformants were spotted onto double dropout (DDO; SD/−Leu/−Trp) and quadruple dropout (QDO; SD/−Ade/−His/−Leu/−Trp) media. (B) Validation of the interaction by in vitro pull‐down assay. (C) BiFC assays showing that BnaA07.NADK3 interacts with BnaA03.PEX5 within peroxisomes of *N. benthamiana* epidermal cells. Scale bars = 20 μm. (D) LCI assays detected luminescence exclusively in sector ④ co‐expressing both proteins, while it was absent in the empty vector controls ①–③. Scale bars = 2 cm.

### BnaC08.WHY1 directly binds to the *BnaA07.NADK3* promoter and is involved in its transcriptional regulation

To identify upstream transcription factors regulating *BnaA07.NADK3*, we employed DNA pull‐down coupled with mass spectrometry (MS) technology and screened a set of candidate proteins (Table S2). Among these, WHY1 was selected for further analysis due to its established association with ROS‐related pathways in previous studies (Foyer et al., 2014; Taylor et al., 2023; Zhuang et al., 2019), leading us to hypothesize that BnaC08.WHY1 may transcriptionally regulate *BnaA07.NADK3*. Bioinformatics analysis of the *BnaA07.NADK3* promoter revealed multiple potential binding elements for BnaC08.WHY1 (Figure 6A). Based on the distribution of these elements, we generated three truncated promoter fragments of *BnaA07.NADK3* (P1: 522 bp, P2: 486 bp, and P3: 364 bp) and conducted yeast one‐hybrid (Y1H) assays, which showed that BnaC08.WHY1 could bind to all three truncated fragments (Figure 6B).

**Figure 6:**
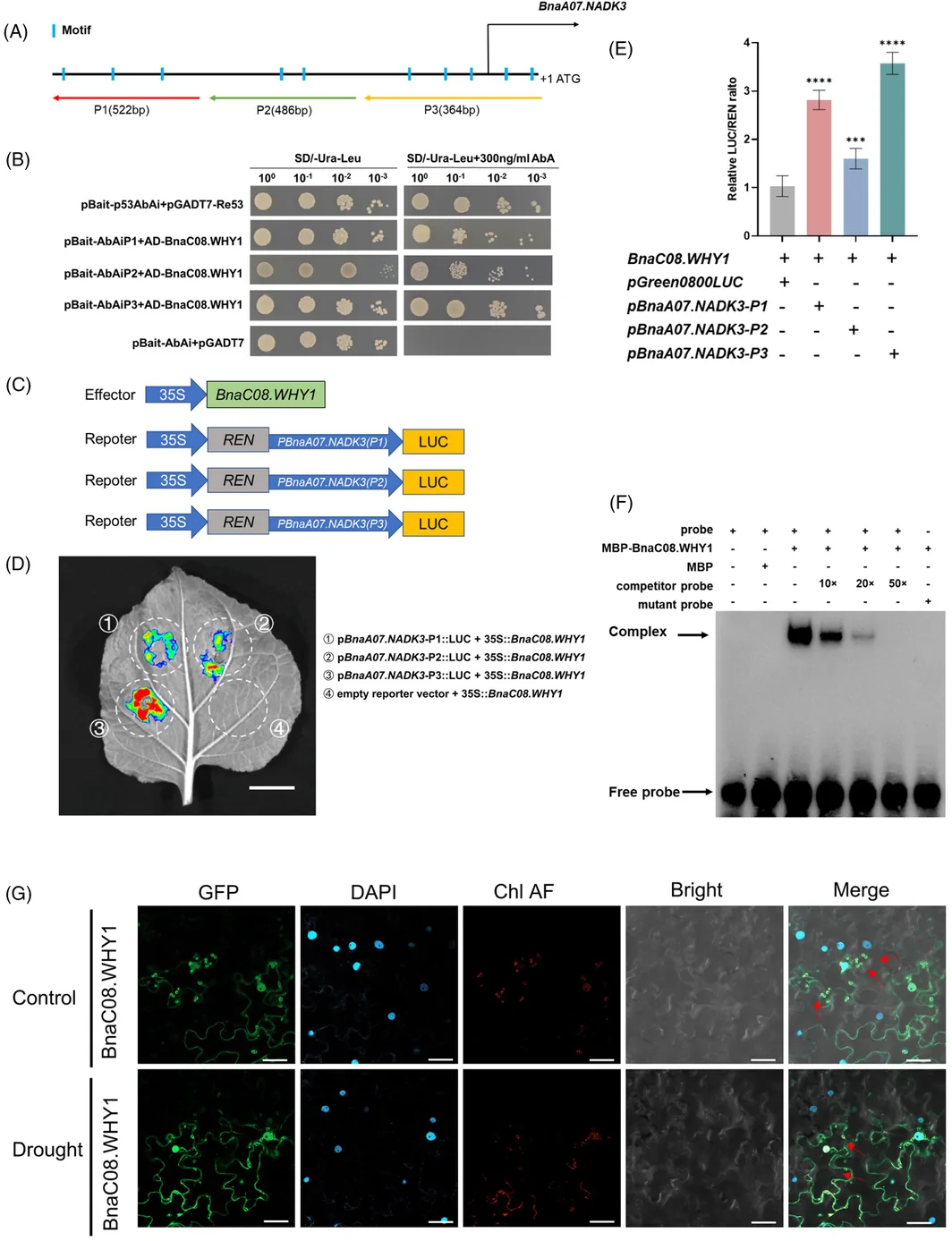
BnaC08.WHY1 binds to the *BnaA07.NADK3* promoter and activates its expression. (A) Schematic diagram of the *BnaA07.NADK3* promoter showing putative BnaC08.WHY1‐binding elements (highlighted in blue). (B) Y1H assay confirming the interaction between BnaC08.WHY1 and three truncated promoter fragments of *BnaA07.NADK3*. (C) Schematic representation of effector and reporter constructs used in dual‐luciferase assays. (D) Dual‐luciferase reporter assay in *N. benthamiana* leaves using BnaC08.WHY1 as the effector and truncated *BnaA07.NADK3* promoter fragments. Scale bars = 2 cm. (E) Quantification of LUC/REN ratio indicating relative transcriptional activity. Data represent means ± SD from three independent biological replicates. Statistical significance was determined by Student's *t*‐test (***P < 0.001, ****P < 0.0001). (F) EMSA demonstrating direct binding of BnaC08.WHY1 to the TTTTTGAC element within the P3 region of the *BnaA07.NADK3* promoter. ‘+’ and ‘−’ indicate presence or absence of reaction components. (G) Subcellular localization of BnaC08.WHY1–GFP under control and drought conditions in *N. benthamiana* epidermal cells. GFP, DAPI‐stained nuclei, and chlorophyll autofluorescence are shown in green, blue, and red, respectively. Scale bars = 50 μm.

Dual‐luciferase reporter assays were conducted using constructs expressing *BnaC08.WHY1* as the effector and plasmids containing the truncated promoter fragments as reporters. All three truncated fragments exhibited higher LUC/REN ratios compared to the negative control, with the P3 fragment conferring the highest ratio, indicating that BnaC08.WHY1 binds to the *BnaA07.NADK3* promoter and activates its expression (Figure 6C–E). Electrophoretic mobility shift assay (EMSA) further confirmed that BnaC08.WHY1 directly binds to the TTTTTGAC element located within the P3 region (Figure 6F). Together, these results indicate that BnaC08.WHY1 associates with the *BnaA07.NADK3* promoter and functions as a transcriptional activator.

We next examined the subcellular localization of BnaC08.WHY1 under normal and drought stress conditions. In tobacco epidermal cells, BnaC08.WHY1‐GFP fluorescence was detected in both chloroplasts and the nucleus under normal growth conditions, a characteristic typical of WHIRLY family proteins (Ren et al., 2017). However, following 48 h of drought treatment, a marked redistribution was observed: chloroplastic signals decreased while nuclear signals became more pronounced (Figure 6G). This shift suggests that BnaC08.WHY1 undergoes stress‐induced translocation from chloroplasts to the nucleus under drought conditions. Previous studies have proposed that such relocalization may be triggered by drought‐induced ROS accumulation in chloroplasts (Li & Kim, 2022). Upon nuclear translocation, BnaC08.WHY1 likely functions as a transcription factor that regulates downstream stress‐responsive genes, including *BnaA07.NADK3*, thereby integrating chloroplast‐derived oxidative signals with nuclear transcriptional processes to enhance antioxidant capacity and drought tolerance in rapeseed.

### The WHY1–NADK3–PEX5 module maintains root growth and ROS homeostasis under stress

To genetically dissect the functional interplay among *WHY1*, *NADK3*, and *PEX5* in stress adaptation, we generated *nadk3 why1* and *nadk3 pex5* double mutants (Figure S2B) in *Arabidopsis* and analyzed their phenotypic responses to 10 μm ABA and 200 mm mannitol separately (Figure 7A,B). Under control conditions, primary root lengths of all double and single mutants were comparable to those of the wild type. However, under ABA and mannitol treatments, the *nadk3 why1* and *nadk3 pex5* double mutants displayed markedly more inhibited primary root elongation in comparison to the corresponding single mutants, while the single mutants themselves showed reduced root growth relative to WT (Figure 7C,D). This progressive phenotypic enhancement from single to double mutants suggests potential synergistic genetic interactions among these three components in stress‐responsive root growth regulation.

**Figure 7:**
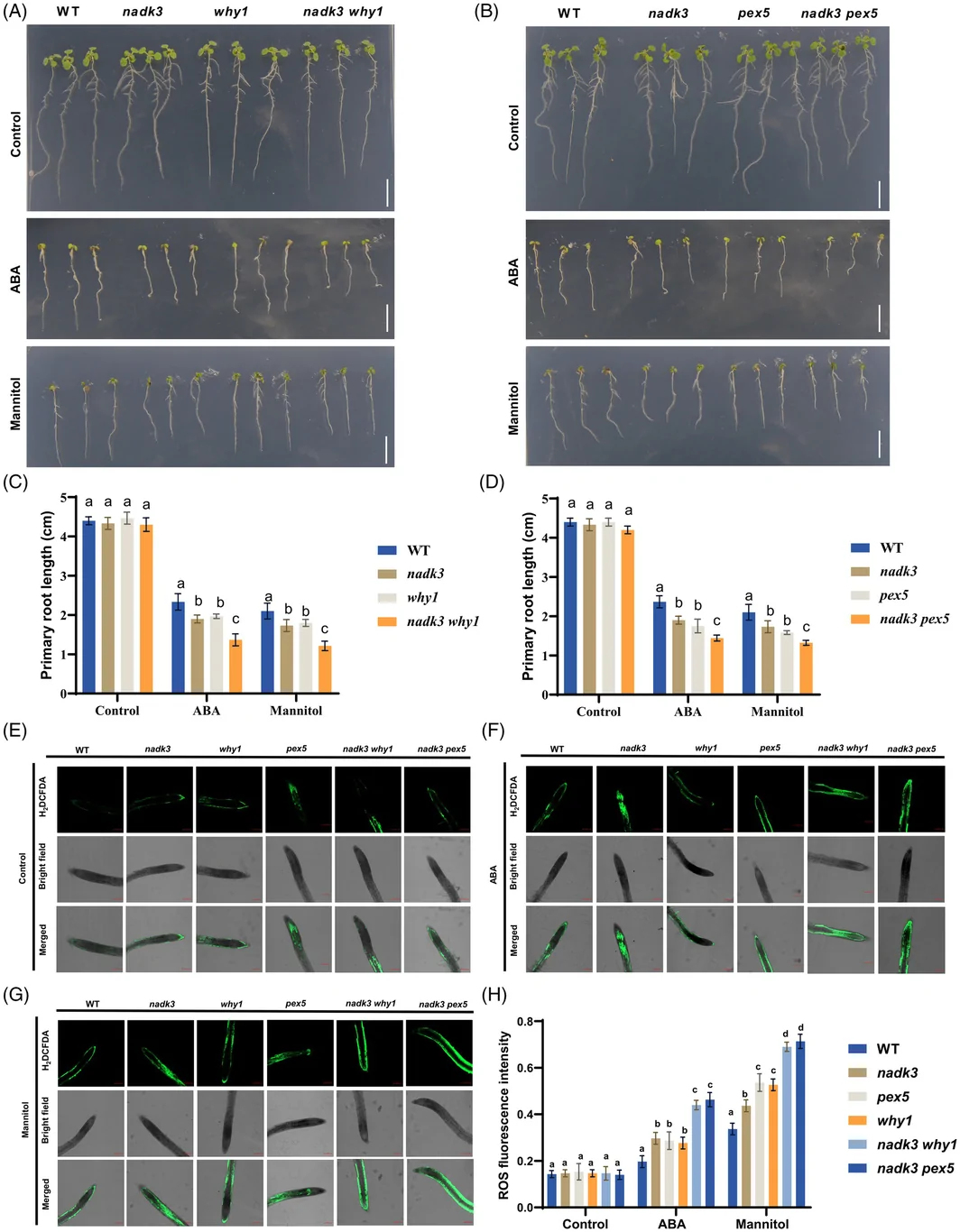
The WHY1–NADK3–PEX5 module is essential for root growth and ROS homeostasis under abiotic stress. (A, B) Representative phenotypes of WT, single mutants (*nadk3*, *why1*, *pex5*), and double mutants (*nadk3 why1*, *nadk3 pex5*) grown on 1/2 MS medium under control, 10 μm ABA, or 200 mm mannitol conditions for 7 days. Scale bars = 1 cm. (C, D) Quantitative analysis of primary root lengths for the indicated genotypes in A and B. (E–G) Confocal laser scanning micrographs of H_2_DCFDA‐stained root apices from WT, single and double mutants under control, ABA, and mannitol treatments, showing ROS accumulation. Scale bars = 100 μm. (H) Quantification of relative fluorescence intensity from H_2_DCFDA staining. Data are presented as mean ± SD (*n* = 3 biological replicates; 15 plants per genotype per replicate). Different lowercase letters indicate significant differences among genotypes within the same treatment (one‐way anova followed by Tukey's test, *P* < 0.05).

To determine whether these growth defects were associated with ROS metabolism, we performed H_2_DCFDA staining to visualize and quantify ROS levels in root apices. Under control conditions, no significant differences in ROS accumulation were observed among genotypes. However, following ABA or mannitol treatment, both double mutants displayed markedly higher fluorescence intensity compared to all single mutants and WT, indicating excessive ROS accumulation (Figure 7E–H). This heightened oxidative stress correlates with the observed root growth inhibition and suggests that disruption of the WHY1–NADK3–PEX5 module compromises cellular redox homeostasis under ABA and mannitol conditions. Taken together, these genetic data suggest that WHY1, NADK3, and PEX5 function within a shared stress response network that regulates root elongation and ROS homeostasis under stress.

## DISCUSSION

### *BnaA07.NADK3* enhances drought tolerance by maintaining redox homeostasis

As the sole enzyme catalyzing the phosphorylation of NAD(H) to NADP(H) (Chen et al., 2024), NADK plays important roles in plant growth, development, and stress responses across diverse species (Feitosa‐Araujo et al., 2022; Ji et al., 2022; Suzuki et al., 2023; Wang, Liu, Qin, et al., 2024). For example, the Arabidopsis *nadk3* mutant shows increased sensitivity to ABA and abiotic stresses, including NaCl and mannitol (Chai et al., 2006). However, the contributions of individual NADK members to stress signaling and redox regulation remain poorly understood, particularly in rapeseed.

Our results identify *BnaA07.NADK3* as a positive regulator of drought tolerance and a modulator of ABA responses in rapeseed. Overexpression of *BnaA07.NADK3* enhances drought tolerance and alleviates ABA‐induced growth inhibition. Under stress conditions, the overexpression lines maintain higher NADK activity and NADPH levels than WT plants and accumulate markedly less ROS, whereas the CRISPR/Cas9‐edited lines show reduced redox‐buffering capacity. Notably, *BnaA07.NADK3* expression was induced during the early response to ABA and mannitol, whereas total NADK activity declined after prolonged treatment. This difference likely reflects the distinct sampling times and measurement levels, because transcript analysis examines a single NADK gene, whereas the enzymatic assay reflects the combined activity of multiple NADK isoforms. The induction of *BnaA07.NADK3* may therefore represent a compensatory response, consistent with the smaller decline in NADK activity in the overexpression lines and the greater decline in the edited lines. Although NADPH availability may affect ROS‐scavenging systems, including SOD, POD, and CAT, whether *BnaA07.NADK3* influences these enzyme activities directly or through broader changes in cellular redox status remains unclear. Together, these findings indicate that *BnaA07.NADK3* promotes drought tolerance by maintaining cellular redox homeostasis and represents a potential target for improving stress tolerance in crops.

### BnaA07.NADK3 associates with BnaA03.PEX5 and contributes to redox regulation during drought

Peroxisomes are important sites of ROS metabolism and require adequate reducing power to maintain redox balance under stress (Walker et al., 2025; Wang, Liu, Han, et al., 2024). In Arabidopsis, NADK3 localizes to the peroxisomal matrix and preferentially phosphorylates NADH, whereas loss of NADK3 reduces NADPH availability under oxidative stress and increases stress sensitivity (Chai et al., 2006; Turner et al., 2005; Waller et al., 2010). The contrasting drought and redox phenotypes of the BnaA07.NADK3 overexpression and gene‐edited lines suggest that this function is conserved in rapeseed and becomes more important as the demand for reducing power increases during drought.

The interaction between BnaA07.NADK3 and BnaA03.PEX5 suggests that PEX5‐mediated targeting may be important for the peroxisomal function of BnaA07.NADK3. Whether drought affects its import into peroxisomes or regulates its activity after import, however, remains to be determined. NADPH provides reducing equivalents for the regeneration of ascorbate and glutathione (Foyer & Kunert, 2024), whereas redox homeostasis depends on the balance and turnover of the NADP(H) pool rather than on NADPH abundance alone (Noctor et al., 2006). The higher NADPH levels in the overexpression lines may therefore indicate a greater capacity to sustain reducing power during drought, rather than simple NADPH accumulation. As neither NADP^+^ nor the peroxisomal NADP(H) pool was quantified, whether BnaA07.NADK3 influences the NADP^+^/NADPH balance remains unclear. These findings are consistent with a role for BnaA07.NADK3 in maintaining peroxisomal redox homeostasis during drought, with BnaA03.PEX5 potentially contributing to its peroxisomal targeting.

### Dual localization of BnaC08.WHY1 under drought coordinates ROS homeostasis

WHIRLY1 has been reported to localize to both chloroplasts and nuclei in several plant species (Isemer et al., 2012; Krupinska et al., 2022; Lin et al., 2020). BnaC08.WHY1 showed a similar dual localization under control conditions, whereas its nuclear accumulation increased during drought. This redistribution is consistent with the proposed involvement of WHIRLY1 in plastid‐to‐nucleus redox signaling (Foyer et al., 2014). Nuclear WHIRLY proteins also participate in stress‐related transcriptional regulation. In cassava, MeWHY proteins bind the PB element in the *MeNCED1* promoter and promote ABA biosynthesis (Yan et al., 2020), whereas barley WHIRLY1 affects drought‐responsive gene expression and associated histone modifications (Janack et al., 2016; Manh et al., 2023).

The regulation of *BnaA07.NADK3* by BnaC08.WHY1 provides a possible link between stress signaling and redox metabolism. BnaC08.WHY1 bound the TTTTTGAC element in the *BnaA07.NADK3* promoter and promoted its transcriptional activity. This sequence is the reverse complement of the PB core motif GTCAAAAA, originally characterized within the elicitor response element of the potato *PR‐10a* promoter (Desveaux et al., 2004). Drought‐associated nuclear accumulation of BnaC08.WHY1 may thus couple stress signaling with the transcriptional regulation of a gene encoding a peroxisomal NADK, thereby contributing to redox adjustment during drought.

### The WHY1–NADK3–PEX5 module contributes to ROS homeostasis and root growth under stress

Drought stress induces substantial remodeling of root architecture, with ABA playing an important role in coordinating adaptive root growth under water limitation (Karlova et al., 2021; Teng et al., 2023). ROS are integral to this response: controlled production supports stress signaling, whereas excessive accumulation causes oxidative damage and restricts growth (Wang, Liu, Han, et al., 2024). The preferential induction of *BnaA07.NADK3* in roots may therefore help sustain the reducing capacity required to limit excessive ROS accumulation while maintaining root growth during prolonged stress.

Taken together, these findings suggest that WHY1, NADK3, and PEX5 may act together to regulate ROS homeostasis in roots under stress. The strong induction of *BnaA07.NADK3* in stressed roots may reflect an adjustment in NADPH supply as the demand for reducing power increases. The interaction between BnaA07.NADK3 and BnaA03.PEX5 further suggests that PEX5 may support the function of BnaA07.NADK3 in peroxisomes. Under control conditions, the *Arabidopsis* mutants showed only minor phenotypic differences. Following ABA or mannitol treatment, however, the *nadk3 why1* and *nadk3 pex5* double mutants displayed stronger inhibition of primary root growth and greater ROS accumulation. These stress‐dependent phenotypes indicate that the functional relationship among WHY1, NADK3, and PEX5 becomes more important as the demand for redox buffering increases and suggest that their contributions are at least partly non‐redundant. Based on the available evidence, BnaC08.WHY1 may regulate *BnaA07.NADK3* at the transcriptional level, whereas BnaA03.PEX5 may help BnaA07.NADK3 function in peroxisomes. Coordination between these processes may limit excessive ROS accumulation and alleviate the inhibition of primary root growth under stress. The genetic relationships among WHY1, NADK3, and PEX5 remain to be resolved. It also remains unclear whether BnaA03.PEX5 affects the peroxisomal import of BnaA07.NADK3 or its activity after import.

In summary, our results support a working model in which drought is associated with increased nuclear accumulation of BnaC08.WHY1, which directly promotes *BnaA07.NADK3* transcription. BnaA03.PEX5 may in turn facilitate the peroxisomal function of BnaA07.NADK3, thereby contributing to NADPH‐dependent redox buffering under stress. Together, these findings identify the WHY1–NADK3–PEX5 module as a potential link between plastid‐to‐nucleus signaling, peroxisomal redox regulation and drought tolerance in *B. napus* and highlight its potential relevance for improving drought resilience in rapeseed (Figure 8).

**Figure 8:**
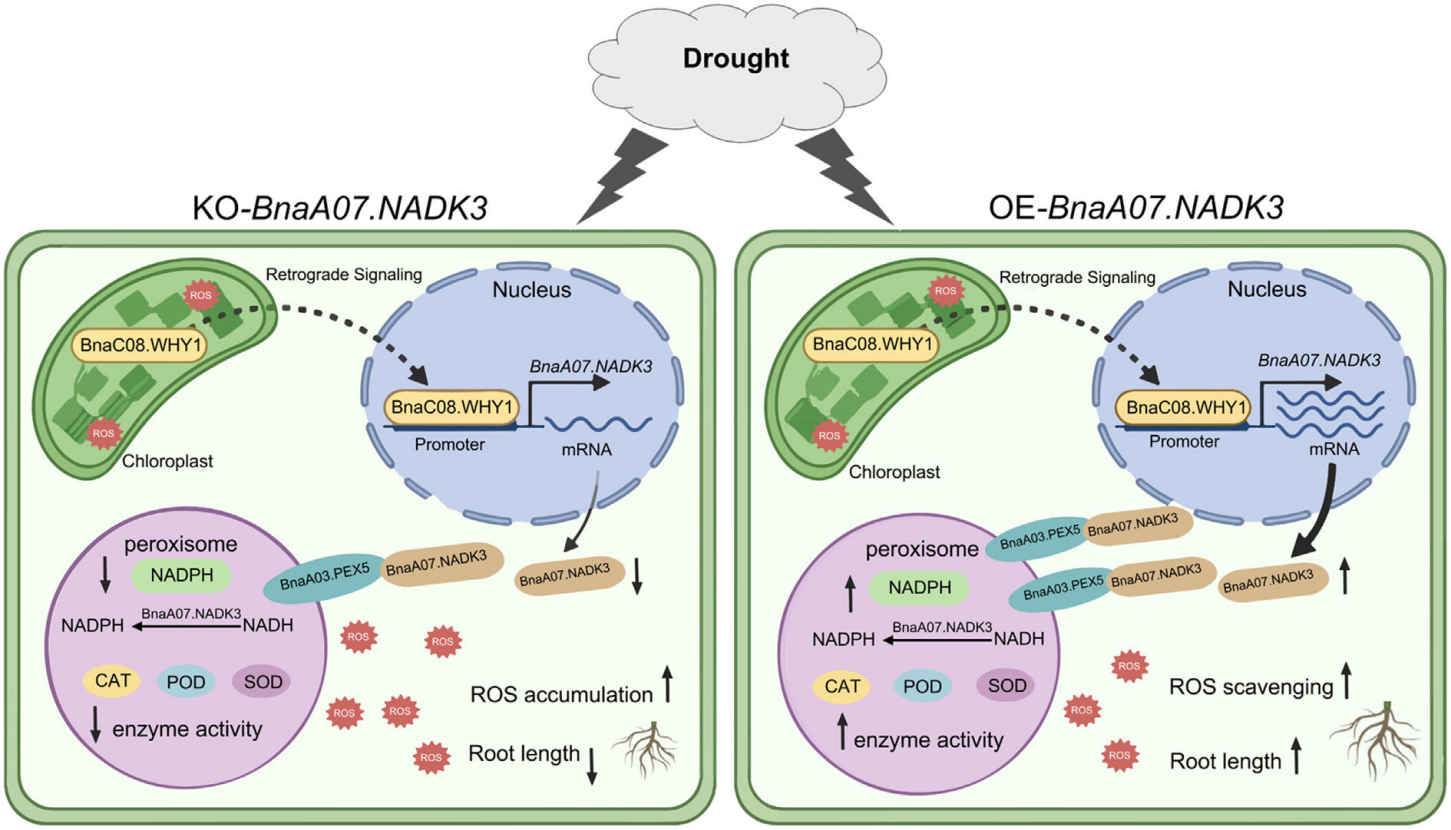
Proposed model for the BnaC08.WHY1–BnaA07.NADK3–BnaA03.PEX5 module in redox regulation and drought tolerance in *B. napus*. Under drought conditions, the nuclear accumulation of BnaC08.WHY1 increases, allowing it to bind directly to the *BnaA07.NADK3* promoter and promote transcription. In the *BnaA07.NADK3*‐edited lines shown on the left, loss of BnaA07.NADK3 function results in lower NADK activity and NADPH content, reduced SOD, POD, and CAT activities, increased ROS accumulation, and inhibited root growth. In the overexpression lines shown on the right, increased BnaA07.NADK3 abundance is associated with higher NADK activity and NADPH content, enhanced antioxidant enzyme activities, reduced ROS accumulation, and improved root growth under stress. The interaction between BnaA07.NADK3 and BnaA03.PEX5 suggests that PEX5 may facilitate the function of BnaA07.NADK3 in peroxisomes. Dashed arrows indicate proposed drought‐associated plastid‐to‐nucleus signaling that remains to be verified. Upward and downward arrows denote increases and decreases, respectively.

## MATERIALS AND METHODS

### Plant materials and abiotic stress treatments

Seeds of *Brassica napus* cv. ‘ZS11’ were germinated on moist gauze for 7 days and transplanted into Hoagland solution for hydroponic culture. After 3 weeks, seedlings were subjected to abiotic stress treatments. Salt, osmotic, and hormonal stresses were induced by adding 200 mm NaCl, 20% (w/v) PEG6000, and 100 μm ABA to the growth solutions, respectively. Additionally, heat and cold treatments were administered by transferring plants to 38 and 4°C chambers. At specific time points (0, 3, 6, and 24 h after the onset of treatments), leaf and root samples were collected, immediately quenched in liquid nitrogen, and kept at −80°C (*n* = 3 biological replicates).

### RNA preparation and assays

Total RNA samples, purified by the Plant RNA Kit R6827 (OMEGA, Beijing, China), were reverse‐transcribed into cDNA using the HiScript II System (Vazyme, Nanjing, China). RT‐qPCR was performed using a LightCycler 96 system (40 cycles), normalizing the data against the reference gene *BnaActin7*. Relative expression was calculated using the 2^−ΔΔ*CT*^ method (Livak & Schmittgen, 2001). The specific primer sets, generated using Primer Premier 5.0, are detailed in Table S3.

### Determination of subcellular localization

The coding sequences of *BnaA07.NADK3* and *BnaC08.WHY1* were cloned into pCAMBIA1300 by homologous recombination to generate GFP fusion constructs. For peroxisomal colocalization, GFP–BnaA07.NADK3 was co‐expressed with mKate–SKL, a peroxisomal marker carrying the C‐terminal PTS1 tripeptide SKL. GFP alone was used as a control. The constructs were introduced into *Agrobacterium tumefaciens* strain GV3101. Agrobacterial cultures were resuspended in infiltration buffer and adjusted to an OD_600_ of 0.8. Suspensions carrying the GFP construct and mKate–SKL marker were mixed and infiltrated into the abaxial side of leaves from 4‐ to 6‐week‐old *Nicotiana benthamiana* plants. Fluorescence was examined 48–72 h after infiltration using a Zeiss LSM 980 confocal laser scanning microscope.

### Vector construction, plant transformation and genotyping

The *BnaA07.NADK3* coding sequence was cloned into pCAMBIA1300 under the CaMV 35S promoter for overexpression. For CRISPR/Cas9 editing, sgRNAs designed via CRISPR‐P 2.0 (Liu et al., 2017) were inserted into the BGK01 vector (BIOGLE GeneTech, Hangzhou, Zhejiang, China). Both constructs were introduced into *Brassica napus* cv. ‘7M314’ using *Agrobacterium tumefaciens* GV3101‐mediated hypocotyl transformation. Transgenic lines were identified by PCR, and mutations in the CRISPR/Cas9‐edited lines were characterized using Hi‐TOM sequencing (Liu et al., 2019). Homozygous lines with frameshifts or premature stop codons were selected, and transcript levels were confirmed by RT‐qPCR. *Arabidopsis thaliana* T‐DNA insertion lines *nadk3* (SALK_201706C), *why1* (SALK_023713C), and *pex5* (SALK_090353C) in the Col‐0 background were obtained from AraShare (https://www.arashare.cn/). Homozygous mutants were identified by three‐primer PCR. The *nadk3 why1* and *nadk3 pex5* double mutants were generated by crossing the corresponding homozygous single mutants. The resulting F1 plants were self‐pollinated, and plants homozygous for both mutant alleles were identified in the F2 generation by PCR‐based genotyping. Primer sequences and representative genotyping results are provided in Table S3 and Figure S2, respectively.

### ROS detection and quantification

ROS accumulation and distribution were analyzed using histochemical staining and H_2_DCFDA fluorescence. To determine the tissue localization of H_2_O_2_ and $O_{2}^{\cdot -}$, the samples were subjected to DAB and NBT staining procedures, utilizing commercial kits procured from Coolaber (Beijing, China). Samples were incubated in the dark at 25°C for 1 h, followed by chlorophyll removal through repeated washing with 95% (v/v) ethanol. Images were captured using a Leica DM6 B light microscope, and staining intensity was quantified using ImageJ (Wang et al., 2023). For total ROS detection, intact roots were pre‐incubated in MES–KCl buffer (Zhang et al., 2021) for 30 min and stained with 50 μm 2′,7′‐dichlorodihydrofluorescein diacetate (H_2_DCFDA; Coolaber) in the dark at 25°C for 30 min. Roots were washed three times (5 min each) with fresh buffer. Fluorescence signals were visualized using a Zeiss LSM 980 confocal laser scanning microscope (excitation: 488 nm; emission: 522 nm) (Bai et al., 2023). The mean fluorescence intensity of regions of interest (ROIs) was quantified using ImageJ.

### Physiological indices and enzymatic assays

Soil‐grown *Brassica napus* seedlings (wild‐type and transgenic lines) were cultivated under regulated environmental parameters (22°C; 16/8 h photoperiod; 60% relative humidity). To induce dehydration stress, irrigation was ceased for 21‐day‐old seedlings for 14 days, followed by rewatering for 7 days. Survival rates were calculated based on plant recovery and leaf regreening (*n* > 10; three biological replicates). The concentrations of malondialdehyde (MDA) and NADPH, along with the activities of NADK, POD, SOD, and CAT, were quantified using commercial assay kits according to the manufacturer's instructions, including the MDA Content Assay Kit (BC0025), Coenzyme II NADP(H) Content Assay Kit (BC5205), NADK Activity Assay Kit (BC1030), POD Activity Assay Kit (BC0095), SOD Activity Assay Kit (BC0175), and CAT Activity Assay Kit (BC0205), all from Solarbio (Beijing, China).

### Histochemical assay of GUS activity

To generate this Pro*BnaA07.NADK3::GUS* reporter construct, an approximately 2.0‐kb regulatory region preceding the *BnaA07.NADK3* translation initiation site was isolated via PCR and ligated into the pCAMBIA1391 backbone. This construct was introduced into the *Agrobacterium rhizogenes* strain K599 through heat shock transformation and was utilized to infect the cotyledons of *Brassica napus*, thereby inducing the formation of transgenic hairy roots. Positive roots were excised and incubated with the GUS Staining Kit (SL7160, Coolaber, Beijing), and the staining patterns were documented using a SONY SENSOR Lighttools camera.

### Yeast one‐hybrid (Y1H) and two‐hybrid (Y2H) assays

For Y2H assays, the full‐length CDSs of *BnaA07.NADK3* and *BnaA03.PEX5* were cloned into the pGADT7 and pGBKT7 vectors, respectively. Protein–protein interactions were assessed by growing yeast transformants on double‐dropout (SD/−Leu/−Trp) and quadruple dropout (SD/−Ade/−His/−Leu/−Trp) synthetic deficient agar at 30°C. For Y1H assays, truncated *BnaA07.NADK3* promoter fragments were inserted into the pAbai bait vector, while the BnaC08.WHY1 CDS was cloned into pGADT7. After co‐transformation into Y1HGold competent cells, transformants were selected on SD/−Leu/−Ura plates supplemented with Aureobasidin A (AbA). The agar dishes were maintained at 30°C for 72 h to identify positive interactions.

### Bimolecular fluorescence complementation

The CDSs of *BnaA07.NADK3* and *BnaA03.PEX5* (lacking stop codons) were cloned into the pCAMBIA1300‐cYFP and pCAMBIA1300‐nYFP plasmids, respectively. These recombinant plasmids were transferred into *Agrobacterium tumefaciens* GV3101 strain. Agrobacterial cultures carrying the respective expression cassettes were combined at equal proportions and agroinfiltrated into the leaf tissues of *Nicotiana benthamiana*. Fluorescence signals were visualized 48–72 h post‐infiltration using a Zeiss LSM 980 confocal laser scanning microscope.

### Luciferase complementation imaging

The complete coding sequences of *BnaA07.NADK3* and *BnaA03.PEX5* were ligated into pCAMBIA1300‐nLUC and pCAMBIA1300‐cLUC plasmids, respectively. Following transient expression in *Nicotiana benthamiana*, leaves were sprayed with a 1 mm D‐luciferin solution, and bioluminescent emissions were recorded employing a NightOWL II LB 983 system.

### Dual‐luciferase reporter assay

Transient expression assays were performed in *N. benthamiana*. We generated reporter plasmids by cloning *BnaA07.NADK3* promoter fragments into pGreenII 0800‐LUC, and the effector plasmid by subcloning the *BnaC08.WHY1* coding region into pCAMBIA1300. Following electroporation into *A. tumefaciens* GV3101 (pSoup), mixed cultures were used to infect tobacco leaves. Luminescence signals were recorded 2 days later to calculate LUC/REN ratios.

### Pull‐down assay

Fusion proteins (MBP‐BnaA07.NADK3 and His‐BnaA03.PEX5) were produced in *E. coli* BL21(DE3) and isolated via amylose resin (NEB) or Ni‐NTA agarose (Smart‐Lifesciences). For pull‐down assays, 50 μg of MBP‐tagged bait protein was immobilized on 50 μl of equilibrated amylose resin in PBS (4°C, 2 h, gentle rotation). Following the addition of His‐tagged prey protein (50 μg), the mixture was incubated overnight at 4°C. Beads were rinsed thrice with PBS, and bound proteins were released by boiling in 1× SDS loading buffer. Immunoblotting analysis was performed using anti‐His and anti‐MBP antibodies (Proteintech, Wuhan, Hubei, China) to validate protein interactions.

### Digital library‐based screening of interacting proteins

To identify potential interacting proteins of BnaA07.NADK3, a comprehensive digital protein library for *Brassica napus* was constructed. Genomic coding sequences (CDS) were translated into protein sequences, and their three‐dimensional structures were predicted via homology modeling using SWISS‐MODEL. BnaA07.NADK3 was employed as the bait protein for an initial round of global molecular docking against the library using MEGADOCK‐4.1.4. Candidate interacting pairs were identified based on integrated scores, including total and rPSC scores. The top 200 candidates were subjected to a second round of screening using an AI‐PPI model to filter out low‐confidence interactions. High‐confidence interactors were further modeled and refined using AlphaPulldown and AlphaFold3 (Yu et al., 2023). The final selection of candidates was based on iptm_ptm scores and structural interface analyses conducted in PyMOL.

### Identification of transcription factors binding the BnaA07.NADK3 promoter by DNA pull‐down coupled with LC–MS/MS

Biotin‐labeled primers were designed based on the promoter sequence of *BnaA07.NADK3* and utilized to amplify the probe fragment via PCR. The biotinylated probe was subsequently coupled to streptavidin‐coated magnetic beads, washed, and incubated with total protein extracted from rapeseed at 4°C to enrich for DNA‐binding proteins. Following the washing step, a portion of the sample was used for DNA recovery to verify the probe, while the remaining material was separated by SDS‐PAGE, and the gel regions containing the proteins of interest were excised. The gel pieces underwent a series of processes, including dehydration, reduction, alkylation, and in‐gel trypsin digestion. The resulting peptides were extracted through ultrasonication, concentrated, desalted using C18 cartridges, and analyzed by LC–MS/MS on an Orbitrap Astral mass spectrometer coupled with a Vanquish NEO system operating in DDA mode (Huang et al., 2025). The acquired MS spectra were analyzed using Proteome Discoverer software, leading to the identification of candidate transcription factors that bind to the *BnaA07.NADK3* promoter fragment based on the proteomic results.

### Electrophoretic mobility shift assay (EMSA)

The LightShift Chemiluminescent EMSA Kit (Thermo Fisher Scientific, Waltham, MA, USA) was utilized to perform the electrophoretic mobility shift assays (EMSA). Purified recombinant MBP‐BnaC08.WHY1 was mixed with 5′‐biotinylated dsDNA probes (containing the TTTTTGAC motif) in binding buffer at 25°C for 30 min. For competition assays, unlabeled competitor probes were added as indicated. After electrophoresis on 6% native gels (0.5× TBE, 4°C), samples were transferred to Biodyne® B nylon membranes (Pall, Port Washington, NY, USA) and UV‐crosslinked. Detection was carried out via chemiluminescence using the ChemiDoc XRS system.

### Stress treatments of transgenic *Brassica napus*

Seeds from WT and transgenic rapeseed genotypes underwent surface sterilization with a 75% ethanol wash, and were then treated with 0.15% HgCl_2_. After five rinses with sterile distilled water, the seeds were air‐dried and then sown on 1/2 Murashige and Skoog (MS) agar medium. Following a 2‐day germination period, the seedlings were transferred to vertically oriented half‐strength MS plates supplemented with either 100 μm ABA or 200 mm mannitol. Root growth data were collected after 7 days. Soil‐grown plants (started from 5‐day‐old seedlings) were kept at a constant 22°C environment (16‐h light/8‐h dark cycle, 60% relative humidity). Drought stress was initiated by water deprivation for 14 days after 3 weeks of normal growth.

### Statistical analysis

Data are presented as means ± SD from at least three independent biological replicates unless otherwise stated. Statistical analyses were performed using GraphPad Prism 9 (GraphPad Software, Boston, MA, USA). Two‐tailed unpaired Student's *t*‐tests were used for comparisons between two groups. One‐ or two‐way anova was used for experiments involving one or two factors, respectively. Dunnett's test was used for comparisons with a single control, whereas Tukey's test was used for all pairwise comparisons. *P* < 0.05 was considered statistically significant. Specific tests are indicated in the corresponding figure legends.

## AUTHOR CONTRIBUTIONS

XD and HW conceived the project. XD supervised the project and modified the paper. JY, ZQ, CW, ZT, LK, and YM performed the experiments. JY analyzed the data and wrote the paper. Each of the contributors participated in preparing the current work and consented to its submitted form.

## CONFLICT OF INTEREST

The authors declare that they have no competing interests.

## Supporting information


**Figure S1.** Phylogenetic analysis and expression profiling of the *NADK* gene family in *Brassica napus*.
**Figure S2.** Characterization of *BnaA07.NADK3* CRISPR/Cas9‐edited lines, overexpression lines, and *Arabidopsis* mutants.
**Figure S3.** BnaA07.NADK3 positively regulates ABA and drought tolerance *in B. napus*.
**Figure S4.** Expression of ABA‐responsive genes in *BnaA07.NADK3* CRISPR/Cas9‐edited and overexpression lines.
**Figure S5.** Transcriptomic analysis of commonly downregulated genes in *BnaA07.NADK3*‐overexpressing and WT plants under ABA and mannitol treatments.
**Figure S6.** Heatmap and RT–qPCR analyses of redox‐ and antioxidant‐related genes in *BnaA07.NADK3* overexpression lines.
**Figure S7.** *BnaA07.NADK3* overexpression reduces ROS accumulation under ABA and mannitol stress.
**Figure S8.** ROS accumulation in leaves of soil‐grown *Brassica napus* plants subjected to soil drying.
**Figure S9.** Coomassie Brilliant Blue‐stained SDS‐PAGE analysis of the recombinant proteins used in the pull‐down assay.
**Table S1.** Potential interaction partners of BnaA07.NADK3 identified through electronic library‐based screening.
**Table S2.** Candidate upstream transcription factors of *BnaA07.NADK3* identified by DNA pull‐down coupled with LC–MS/MS.
**Table S3.** Primers used in this study.

## Data Availability

The datasets generated and analyzed during the current study are available within the article and its supplementary materials. Furthermore, the raw RNA‐seq reads produced in this research have been archived in the National Center for Biotechnology Information (NCBI) database under the BioProject accession number PRJNA1431922.
