# Supplementary material for: A WHY1–NADK3–PEX5 module confers drought tolerance by maintaining peroxisomal ROS homeostasis in Brassica napus
Source: Plant J. 2026 Sep 30;127(6):e71124. doi: 10.1111/tpj.71124 (PMC13627963; doi:10.1111/tpj.71124)
Supplement: Supplementary file 1 [file TPJ-127-0-s001.docx]

**SUPPORTING INFORMATION**

**Figure S1.** Phylogenetic analysis and expression profiling of the *NADK* gene family in *Brassica napus*.

**Figure S2.** Characterization of *BnaA07.NADK3* CRISPR/Cas9-edited lines, overexpression lines, and *Arabidopsis* mutants.

**Figure S3.** *BnaA07.NADK3* positively regulates ABA and drought tolerance *in B. napus*.

**Figure S4.** Expression of ABA-responsive genes in *BnaA07.NADK3* CRISPR/Cas9-edited and overexpression lines.

**Figure S5.** Transcriptomic analysis of commonly downregulated genes in *BnaA07.NADK3*-overexpressing and WT plants under ABA and mannitol treatments.

**Figure S6.** Heatmap and RT–qPCR analyses of redox- and antioxidant-related genes in *BnaA07.NADK3* overexpression lines.

**Figure S7.** *BnaA07.NADK3* overexpression reduces ROS accumulation under ABA and mannitol treatments.

**Figure S8.** ROS accumulation in leaves of soil-grown *B. napus* plants subjected to soil drying.

**Figure S9.** Coomassie Brilliant Blue-stained SDS-PAGE analysis of the recombinant proteins used in the pull-down assay.

**Table S1.** Candidate BnaA07.NADK3-interacting proteins identified by digital protein library-based screening.

**Table S2.** Candidate upstream transcription factors of *BnaA07.NADK3* identified by DNA pull-down coupled with LC–MS/MS.

**Table S3.** Primers used in this study.

**Figure S1.** **Phylogenetic analysis and expression profiling of the *NADK* gene family in *Brassica napus*.** (A) Phylogenetic tree of NADK proteins from *Arabidopsis thaliana* (red triangles), *Brassica napus* (blue circles), *Brassica rapa* (purple stars), and *Brassica oleracea* (green squares). The NADK members are clustered into three distinct subfamilies (Group 1, Group 2, and Group 3). (B) Heatmap showing the spatio-temporal expression patterns of 12 *BnaNADK* genes in leaves (L, cyan bar) and roots (R, pink bar). Transcript levels were assessed under control conditions (CK), hormone treatment (ABA) and various abiotic stresses (NaCl, PEG, Cold, and Heat) at 0, 3, 6, and 24 h. The color scale represents normalized transcript abundance [log_2_ (average+1)], ranging from blue (low) to red (high). *BnaA07.NADK3* is highlighted in red and was further extracted for the focused analysis shown in Fig. 1A.

**Figure S2. Characterization of *BnaA07.NADK3* CRISPR/Cas9-edited lines, overexpression lines, and *Arabidopsis* mutants. (A)** Schematic representation of the BnaA07.NADK3 gene structure and the CRISPR/Cas9 target site. Blue boxes indicate exons, black lines indicate introns, and the arrow indicates the 5′ to 3′ orientation of the gene. The sgRNA target sequence and PAM site are shown below the gene model. Mutated nucleotides in the CRISPR/Cas9-edited lines KO-1 and KO-2 are highlighted in red. The corresponding predicted amino acid sequences are shown on the right, with premature stop codons indicated by asterisks. **(B)** PCR genotyping of the *Arabidopsis* *why1*, *nadk3*, *pex5*, *nadk3 why1*, and *nadk3 pex5* mutants. LP/RP primer pairs amplified the wild-type alleles, whereas BP/RP primer pairs amplified the T-DNA insertion alleles. **(C)** RT-qPCR analysis of BnaA07.NADK3 transcript levels in CRISPR/Cas9-edited and overexpression lines. Expression levels were normalized to the internal reference gene. Data are presented as mean ± SD (n = 3). Asterisks indicate significant differences from WT according to Student’s t-test: ^*^*P* < 0.05, ^**^*P* < 0.01, ^***^*P* < 0.001,^**^^**^*P* < 0.0001.

**Figure S3. BnaA07.NADK3 positively regulates ABA and drought tolerance in *B. napus*.** (A) Phenotypic comparison of WT and overexpression (OE-2, OE-5), and CRISPR/Cas9-edited lines (KO-1, KO-2) seedlings grown on 1/2 MS medium supplemented with 100 μM ABA or 200 mM mannitol for 7 days. Scale bars = 1 cm. (B,C) Primary root length and biomass for each genotype under indicated treatments. (D–F) Activities of catalase (CAT, D), superoxide dismutase (SOD, E), and peroxidase (POD, F). (G) Malondialdehyde (MDA) content. Data are presented as mean ± SD (n = 3 biological replicates; 15 plants per genotype per replicate). Data were analyzed by one-way ANOVA followed by Dunnett’s multiple comparisons test. Asterisks indicate significant differences compared with WT under the same treatment (^*^*P* < 0.05, ^**^*P* < 0.01, ^***^*P* < 0.001).

**Figure S4. Expression of ABA-responsive genes in *BnaA07.NADK3* CRISPR/Cas9-edited and overexpression lines.** (A) RT-qPCR analysis of *RAB18* (*BnaA05G0189900ZS*), *RD29B* (*BnaC02G0186200ZS*), *NCED3* (*BnaA01G0353200ZS*), and *RD22* (*BnaA06G0338200ZS*) in WT, *BnaA07.NADK3* CRISPR/Cas9-edited lines, and *BnaA07.NADK3* overexpression lines under control, ABA, and mannitol treatments. Expression levels were normalized to the internal reference gene. Data are means ± SD from three biological replicates. Asterisks indicate significant differences from WT under the same treatment according to Student’s *t*-test: ^*^*P* < 0.05, ^**^*P* < 0.01, ^***^*P* < 0.001,^****^*P*<0.0001.

**Figure S5. Transcriptomic analysis of commonly down-regulated genes in *BnaA07.NADK3* overexpression and WT plants under ABA and mannitol treatments**. (A, B) Venn diagrams showing the overlap of down-regulated differentially expressed genes (DEGs) across different treatments at 6 h (A) and 1 week (B). The red highlighted regions indicate the commonly down-regulated DEGs under both stress conditions. (C, D) KEGG pathway (C) and GO (D) enrichment analyses of the 1453 commonly down-regulated DEGs at 6 h. (E, F) KEGG pathway (E) and GO (F) enrichment analyses of the 73 commonly down-regulated DEGs at 1 week. BP, biological process; CC, cellular component; MF, molecular function.

**Figure S6. Heatmap and RT–qPCR analyses of redox- and antioxidant-related genes in *BnaA07.NADK3* overexpression lines.** (A–D) Heatmaps showing log₂ fold changes of differentially expressed genes between overexpression (OE) and wild-type (WT) plants under control, ABA, and mannitol treatments. The genes shown are enriched in heme binding, oxidoreductase activity, ascorbate and aldarate metabolism, and peroxidase-related pathways. (E–K) RT–qPCR validation of representative genes in WT and OE plants under the corresponding treatment conditions. Data represent means ± SD of three independent biological replicates. Data were analyzed by one-way ANOVA followed by Dunnett’s multiple comparisons test. Asterisks indicate significant differences compared with WT under the same treatment ^(*^*P* < 0.05, ^**^*P* < 0.01, ^***^*P* < 0.001, ^****^*P* < 0.0001).

**Figure S7. *BnaA07.NADK3* overexpression reduces ROS accumulation under ABA and mannitol treatments.** (A–D) NBT and DAB staining assays of leaves (A, B) and root tips (C, D) from WT, BnaA07.NADK3 OE and CRISPR/Cas9-edited lines under control conditions or after treatment with 100 µM ABA or 200 mM mannitol (Scale bars = 0.5 cm). (E–H) Quantitative analysis of NBT and DAB staining intensities in leaves (E, F) and roots (G, H). Data are presented as mean ± SD (n = 3 biological replicates; 5 plants per genotype per replicate). Data were analyzed by one-way ANOVA followed by Dunnett’s multiple comparisons test. Asterisks indicate significant differences compared with WT under the same treatment (^*^*P*< 0.05, ^**^*P* < 0.01, ^***^*P* < 0.001).

**Figure S8. ROS accumulation in leaves of soil-grown *B. napus* plants subjected to soil drying.** (A, B) Representative images of NBT (A) and DAB (B) staining in leaves of WT, *BnaA07.NADK3* CRISPR/Cas9-edited lines (KO-1 and KO-2), and overexpression lines (OE-2 and OE-5) under well-watered and soil-drying conditions. Scale bars = 2 cm. NBT and DAB staining were used to detect superoxide (O_2_^•⁻^) and hydrogen peroxide (H₂O₂), respectively. (C, D) Average optical density of NBT (C) and DAB (D) staining quantified using ImageJ. Data are presented as mean ± SD (n = 3 biological replicates; 5 plants per genotype per replicate). Asterisks indicate significant differences from WT under the same treatment, as determined by one-way ANOVA followed by Dunnett’s multiple-comparison test (^*^*P* < 0.05, ^**^*P* < 0.01 and ^***^*P* < 0.001).

**Figure S9. Coomassie Brilliant Blue-stained SDS-PAGE analysis of the recombinant proteins used in the pull-down assay.** (A) Purification of His–BnaA03.PEX5. The crude extract and fractions eluted with the indicated concentrations of imidazole are shown. (B) Purification of MBP–BnaA07.NADK3. The crude extract, wash fraction, and fractions eluted with 20 mM maltose are shown. Target protein bands are indicated by red arrows. The predicted molecular masses of His–BnaA03.PEX5 and MBP–BnaA07.NADK3 are approximately 97.9 and 82.9 kDa, respectively.

| **Table S1.**  **Candidate BnaA07.NADK3-interacting proteins identified by digital protein library-based screening** | | | | | |  |
| --- | --- | --- | --- | --- | --- | --- |
| **ID** | **Description** | | **AF3_iptm** | **AF3_ptm** | **AF3_iptm+ptm** | |
| BnaA05G0450700ZS | | F-box Kelch-repeat protein | 0.46 | 0.64 | 1.1 | |
| BnaC09G0240000ZS | | ADP binding | 0.67 | 0.69 | 1.36 | |
| BnaC02G0312900ZS | | ATP-NAD kinase | 0.75 | 0.8 | 1.55 | |
| BnaA03G0117300ZS | | Peroxisome biogenesis protein 5 OS=Arabidopsis thaliana GN=PEX5 PE=1 SV=1 | 0.68 | 0.52 | 1.2 | |
| BnaC09G0505300ZS | | Glycosyl hydrolase family 81 protein | 0.25 | 0.69 | 0.94 | |
| BnaC08G0302900ZS | | Belongs to the peptidase A1 family | 0.33 | 0.47 | 0.8 | |
| BnaA02G0353700ZS | | BAG family molecular chaperone regulator 8, chloroplastic-like | 0.14 | 0.42 | 0.56 | |
| BnaC03G0595400ZS | | Belongs to the TRAFAC class myosin-kinesin ATPase superfamily. Kinesin family | 0.15 | 0.32 | 0.47 | |
| BnaA02G0252500ZS | | isoform X1 | 0.09 | 0.68 | 0.77 | |
| BnaA07G0219400ZS | | Acts in the modification of cell walls via demethylesterification of cell wall pectin | 0.14 | 0.59 | 0.73 | |

**Table S2. Candidate upstream transcription factors of *BnaA07.NADK3* identified by DNA pull-down coupled with LC–MS/MS.**

| **ZS11 Gene ID** | **At Name** | **Functional description** |
| --- | --- | --- |
| BnaA01G0015900ZS | MRG1 | MRG family protein, possibly involved in chromatin regulation |
| BnaA06G0000300ZS | NOP56 | Nucleolar protein involved in rRNA processing and ribosome biogenesis |
| BnaA08G0183900ZS | U2AF65A | Pre-mRNA splicing factor involved in spliceosome assembly |
| BnaC01G0277000ZS | -- | Transcription factor IIIC subunit involved in RNA polymerase III transcription |
| BnaC03G0346200ZS | RAD23C | Protein involved in ubiquitin/26S proteasome-mediated substrate delivery |
| BnaC05G0176400ZS | HMGB2 | HMG-box DNA-binding protein involved in nucleoprotein complex assembly |
| BnaC07G0484700ZS | IAA11 | Auxin-responsive protein involved in auxin signaling |
| BnaC07G0513100ZS | REM16 | AP2/B3-like transcription factor potentially involved in transcriptional regulation |
| BnaC08G0228600ZS | WHY1 | Single-stranded DNA-binding protein involved in chloroplast-to-nucleus retrograde signaling |
| BnaC08G0297100ZS | SR34A | SR-type splicing factor involved in alternative splicing regulation |
| BnaC08G0320300ZS | HMGB1 | HMG-box DNA-binding protein involved in chromatin organization and nuclear structure |
| BnaC09G0122000ZS | U2AF65A | Pre-mRNA splicing factor involved in spliceosome assembly |
| Bnascaffold0027G0055600ZS | RANBP1A | Ran-binding protein involved in nucleocytoplasmic transport |

**Table S3. Primers used in this study**

| **Primer name** | **Sequence 5'-3** | **Description** |
| --- | --- | --- |
| BnaA07.NADK3-OEF | CAAGCTGACTCTAGCAGATCTATGGCGATCAGGAAGCTT | OE |
| BnaA07.NADK3-OER | TCCTTTGCCCATGGCTCTAGACTAGTACCTTGATCTGACCTGACT |  |
| A7CAS-F | gagtacggtgtgcGGAGATACTAAATAAGAAGGCAG | CRISPR/Cas9 |
| A7CAS-R | ggatgctggatggGCTAAAAGACCTCGAGAGCT |  |
| BnaA07.NADK3QDZ-F | TGACTCCATgtcgacggatccGGTTGAGCCAATTACACTTATTAG | Pro*BnaA07.NADK3*::GUS |
| BnaA07.NADK3QDZ-R | ggtggactcctcttagaattcTCTCCTTGAACTCAATCTCTTTAT |  |
| BDBnaA07.NADK3-F | atggccatggaggccgaattcATGGCGATCAGGAAGCTTCTG | Y2H |
| BDBnaA07.NADK3-R | ccgctgcaggtcgacggatccGTACCTTGATCTGACCTGACTAAACC |  |
| ADBnaA07.NADK3-R | CAGCTCGAGCTCGATGGATCCCTAGTACCTTGATCTGACCTGACTAAACC |  |
| ADBnaA07.NADK3-F | GCCATGGAGGCCAGTGAATTCATGGCGATCAGGAAGCTTCTG |  |
| BDWHY1-R | CAGCTCGAGCTCGATGGATCCCTATCTATTCCATTCATAGTCT | Y1H |
| BDWHY1-F | GCCATGGAGGCCAGTGAATTCATGTCGCAACTCTTATCTTCTC |  |
| pAbAi-R | TTTAGAGAAAAGAAGAAAACAAGAG |  |
| pAbAi-F | TATACCAATCTAAGTCTGTGCTCCT |  |
| BnaP3-R | AGCACATGCCTCGAGGTCGACGGTTGAGCCAATTACACTTATTA |  |
| BnaP3-F | AAATGATGAATTGAAAAGCTTCTCGATCTAATTTGTGTGTGCA |  |
| BnaP2-R | AGCACATGCCTCGAGGTCGACGTTAACTACCTCAAGACCACTA |  |
| BnaP2-F | AAATGATGAATTGAAAAGCTTCTGATCTTGAAGTGGGGTTTG |  |
| BnaP1-R | AGCACATGCCTCGAGGTCGACGTTCATCAAAGACATTCGTC |  |

**Table S3. Continued**

| **Primer name** | **Sequence 5'-3** | **Description** |
| --- | --- | --- |
| BnaP1-F | AAATGATGAATTGAAAAGCTTCTCCTTGAACTCAATCTCTTT | Y1H |
| BFBnaA07.NADK3-F | aaatacacacactaatctagaATGGCGATCAGGAAGCTTCTG | BiFC |
| BFBnaA07.NADK3-R | atggccgaggcggccttaattaaCTAGTACCTTGATCTGACCTGACTAAACC |  |
| BFBnaA03.PEX5-F | atcgaggacgccggcggatccATGGCGATGAGAGACCTTGTCA |  |
| BFBnaA03.PEX5-R | gctctgcaggtcgactctagaAAGTGGGAACTCTTTCTGCA |  |
| A03PEX5-LUCF | aacacgggggacgagctcggtaccATGGCGATGAGAGACCTTGTC | LCI |
| A03PEX5-LUCR | gacgcgtacgagatctggtcgacAAGTGGGAACTCTTTCTGC |  |
| A07NADK3-LUCF | cgtacgcgtcccggggcggtaccATGGCGATCAGGAAGCTTCT |  |
| A07NADK3-LUCR | ccttgtagtccatttgttggatccCTAGTACCTTGATCTGACCTGACT |  |
| MC1300PEX5-F | gagaacacgggggactctagaATGGCGATGAGAGACCTTGTCA | Subcellular localization |
| MC1300PEX5-R | gcccttgctcaccattctagaAAGTGGGAACTCTTTCTGCAAGA |  |
| A07(TAG)-F | gagaacacgggggactctagaATGGCGATCAGGAAGCTTCTG |  |
| A07(TAG)-R | gcccttgctcaccattctagaGTACCTTGATCTGACCTGACTAAACC |  |
| 1300-WHY1-R | TCCTTTGCCCATGGCTCTAGATCTATTCCATTCATAGTCTCCTCCAT |  |
| 1300-WHY1-F | CAAGCTGACTCTAGCAGATCTATGTCGCAACTCTTATCTTCTCCTC |  |

**Table S3. Continued**

| **Primer name** | **Sequence 5'-3** | **Description** |
| --- | --- | --- |
| BnaC05G0165600ZS-F | ATGGCTCTCGAAGTCTGC | RT-qPCR |
| BnaC05G0165600ZS-R | GAGATGTTGATCAGATGCATT |  |
| BnaA01G0360400ZS-F | ATGCAGGGTGGTAGGCTTTT |  |
| BnaA01G0360400ZS-R | GAGGAATTCCTAGAGGCGCA |  |
| BnaA09G0491800ZS-F | ATGGCGGAGAAGAGCTTCA |  |
| BnaA09G0491800ZS-R | CACCGCCTCTTTGGAGAT |  |
| BnaC06G0309100ZS-F | ATCTGTATCCCATGCTTTTC |  |
| BnaC06G0309100ZS-R | AGTATGGCTGCATTCCTTGG |  |
| BnaA04G0257200ZS-F | ATGGAGATCATCAAGAGCTT |  |
| BnaA04G0257200ZS-R | CCGTAATGCTGTTGTGTGTT |  |
| BnaC07G0468100ZS-F | ATGAGGTCAATCACAGCTTT |  |
| BnaC07G0468100ZS-R | TGATGCTACGGTCACGGC |  |
| BnaC09G0027600ZS-F | ATGGAAACTTTTTACCTGATCG |  |
| BnaC09G0027600ZS-R | TACGGTGTAGCGGCAGCTTT |  |
| BnaA01G0353200ZS-F | ATGGCTTCTTTCACGGCGAAT |  |
| BnaA01G0353200ZS-R | CACGACTTGTCATAGGCATGG |  |
| BnaA06G0338200ZS-F | ATGGCGATTCGTCTTTCGTT |  |
| BnaA06G0338200ZS-R | CGTCGCTAAAATCTGACGTAAAA |  |

**Table S3. Continued**

| **Primer name** | **Sequence 5'-3** | **Description** |
| --- | --- | --- |
| BnaC02G0186200ZS-F | ATCACGAGAGTCGAGCATCTA | RT-qPCR |
| BnaC02G0186200ZS-R | CACATCGTGGTCTTGCTCG |  |
| BnaA05G0189900ZS-F | ATGGGTTCTTCGTCGGGGC |  |
| BnaA05G0189900ZS-R | CACCGATAGTGGGAGAAAGGTC |  |
| BnaActin7-F | CTATCCTCCGTCTCGATCTCGC |  |
| BnaActin7-R | CTTAGCCGTCTCCAGCTCTTGC |  |
| LBb1.3 | ATTTTGCCGATTTCGGAAC | Mutant genotyping |
| nadk3-LP | GTCCGAATTTTGGAGTTAGGC |  |
| nadk3-RP | AATGTTCAAACCAAAATTGCG |  |
| pex5-LP | TTATGGTCCCCTACCTTCGTC |  |
| pex5-RP | CAATCCCGTATTATGTCCGTG |  |
| why1-LP | TGACCAACAAACTGTTGATGG |  |
| why1-RP | TCGAATGACCCACGTAAAATC |  |

**Table S3. Continued**

| **Primer name** | **Sequence 5'-3** | **Description** |
| --- | --- | --- |
| F19061_0S1(+) | gctcacagagaacagattggtgga atggcgatgagagaccttgtcaac | Pull-down |
| F19061_0S1(-) | tttttcaaactgcggatggctcca aagtgggaactctttctgcaagag |  |
| F19061(548C) | catatgacctctagcttgaatgc |  |
| F19061(1096C) | catgcatcagccgaactccc |  |
| F19061(1644C) | gcactctggacactgttgg |  |
| pet30a-F | cggataacaattcccctctag |  |
| pet30a-R | acccctcaagacccgttt |  |
| F19063_0S1(+) | CTCCGGCTCCATTTCACATAatggcgatcaggaagcttctgcttc |  |
| F19063_0S1(-) | CTGCGGATGGCTCCAGAATTctagtaccttgatctgacctgactaaacc |  |
| F19063(477C) | ctcttaggccgctcgagcg |  |
| pMAL-c5x-J99F | gtcgatgaagccctgaaag |  |
| pMAL-c5x-J185R | ccttctgcttaatttgatgcc |  |
| F26047_0S1(+) | CGAGGGAAGGATTTCACATAatggtgaagtctcgccagacgg | EMSA |
| F26047_0S1(-) | TTAATTACCTGCAGGGAATTctatctattccattcatagtctcctccatagttag |  |
| pMAL-c5x-J99F | gtcgatgaagccctgaaag |  |
| pMAL-c5x-J185R | ccttctgcttaatttgatgcc |  |
